# CXCL12/CXCR7/β-arrestin1 biased signal promotes epithelial-to-mesenchymal transition of colorectal cancer by repressing miRNAs through YAP1 nuclear translocation

**DOI:** 10.1186/s13578-022-00908-1

**Published:** 2022-10-09

**Authors:** Mahan Si, Yujia Song, Xiaohui Wang, Dong Wang, Xiaohui Liu, Xianjun Qu, Zhiyu Song, Xinfeng Yu

**Affiliations:** 1 Department of Pharmacology, School of Basic Medical Sciences, Capital Medical University, Beijing, China; 2 Department of General Surgery, Xuanwu Hospital, Capital Medical University, Beijing, China; 3 Department of Pharmacy, Henan Provincial People’s Hospital, People’s Hospital of Zhengzhou University, Zhengzhou, Henan China

**Keywords:** CXCR7, Biased signal, YAP1, miRNAs, Epithelial-to-mesenchymal transition, Colorectal cancer

## Abstract

**Background:**

Chemokine CXC motif receptor 7 (CXCR7) is an atypical G protein-coupled receptor (GPCR) that signals in a biased fashion. CXCL12/CXCR7 biased signal has been reported to play crucial roles in multiple stages of colorectal cancer (CRC). However, the mechanism of CXCL12/CXCR7 biased signal in promoting CRC progression and metastasis remains obscure.

**Results:**

We demonstrate that CXCR7 activation promotes EMT and upregulates the expression of Vimentin and doublecortin-like kinase 1 (DCLK1) in CRC cells with concurrent repression of miR-124-3p and miR-188-5p through YAP1 nuclear translocation. Cell transfection and luciferase assay prove that these miRNAs regulate EMT by targeting Vimentin and DCLK1. More importantly, CXCL12/CXCR7/β-arrestin1-mediated biased signal induces YAP1 nuclear translocation, which functions as a transcriptional repressor by interacting with Yin Yang 1 (YY1) and recruiting YY1 to the promoters of miR-124-3p and miR-188-5p. Pharmacological inhibitor of YAP1 suppresses EMT and tumor metastasis upon CXCR7 activation in vivo in tumor xenografts of nude mice and inflammatory colonic adenocarcinoma models. Clinically, the expression of CXCR7 is positively correlated with nuclear YAP1 levels and EMT markers.

**Conclusions:**

Our studies reveal a novel mechanism and clinical significance of CXCL12/CXCR7 biased signal in promoting EMT and invasion in CRC progression. These findings highlight the potential of targeting YAP1 nuclear translocation in hampering CXCL12/CXCR7 biased signal-induced metastasis of colorectal cancer.

**Supplementary Information:**

The online version contains supplementary material available at 10.1186/s13578-022-00908-1.

## Background

Chemokine CXC motif receptor 4 (CXCR4) has long been thought to be the unique receptor for Chemokine CXC motif ligand 12 (CXCL12) and play important roles in chemotaxis, inflammation, cancer dissemination and organotropic liver metastasis of colorectal cancer (CRC). Until recently, Chemokine CXC motif receptor 7 (CXCR7), an atypical chemokine receptor 3 (ACKR3), was identified to bind to CXCL12 with even higher affinity than CXCR4 [1]. CXCR7 has a dramatic effect on the signaling response resulting from CXCR4 activation because CXCR7 and CXCR4 can form heterodimers, whereby CXCR7 changes the conformation of the CXCR4/G-protein complexes and abrogates their signaling [2]. Although the seven trans-membrane protein CXCR7 could bind to CXCL12, it functions in a G-protein-independent manner. It has been proposed that CXCR7 interacts with β-arrestin (β-arr) for a biased signal transduction as it fails to activate heterotrimeric G proteins. However, much less is known about CXCL12 signaling via CXCR7, which was initially considered to be a sink for CXCL12 [3]. It has been recently reported that CXCR7 interacts with β-arr2 and recruits MAPK proteins for the phosphorylation of ERK [4]. Notably, the serine/threonine residues present at the C-terminal of CXCR7 are essential for β-arr recruitment after receptor activation [5]. Primary structure analysis of CXCR7 indicates it contains the DRYLSIT motif instead of the consensus DRYLAIV motif that is thought to be responsible for receptor/G-protein selectivity and for the efficiency of G-protein activation [6]. However, this CXCR7 motif is also found in other G protein-coupled receptors (GPCRs), suggesting that the motif itself does not explain impaired Gα_i_ activation [7]. Despite the controversy on molecular basis for CXCR7-mediated biased signaling, it is well known that CXCR7 could interact with β-arrestins and signal in a biased manner in response to CXCL12 stimulation [8]. However, it remains unknown how CXCL12/CXCR7 biased signal plays a role in the progression and metastasis of CRC.

Epithelial-to-mesenchymal transition (EMT) is a critical step that initiates metastasis. It refers to epithelial cells acquire the mesenchymal phenotype to facilitate invasion and metastasis. EMT has been linked with stemness, as evidenced by the reactivation of embryonic signaling pathways such as Wnt and Notch [9]. Doublecortin-like kinase 1 (DCLK1), a microtubule associated protein kinase, is overexpressed in colorectal cancer and specifically marks cancer stem cells (CSCs) in the intestine [10]. It has been demonstrated that DCLK1^+^ intestinal cells of *Apc*^Min/+^ mice display higher pluripotency and pro-survival signaling [11]. DCLK1 has been shown to promote EMT and associate with poor prognosis in several cancers [12, 13], suggesting DCLK1 is a potential therapeutic target for cancer invasion and metastasis [14].

The Hippo signaling pathway has emerged as a tumor suppressive pathway that acts to control the transcriptional activity of Yes-associated protein (YAP) and transcriptional coactivator with PDZ-binding motif (TAZ), which plays a central role in regulating EMT plasticity and metastatic potential [15]. YAP/TAZ activity underlies several key hallmarks of cancer through promoting tumor invasion, metastasis and acquisition of CSC characteristics [16]. Activation of the pathway can be triggered by environmental cues such as cell contact, cytoskeletal remodeling, nutrient status and activation of G-protein-coupled receptors [17–19]. Sequential phosphorylation and activation of mammalian STE20-like kinase 1 and 2 (MST1/2) and large tumor suppressor kinases 1/2 (LATS1/2), culminates in the phosphorylation and degradation of the YAP and TAZ. Conversely, when the pathway is deactivated, YAP and TAZ accumulate in the nucleus, associate with DNA-binding proteins, most notably transcriptional enhanced associate domain (TEAD), driving a pro-oncogenic transcriptional program [20].

CXCR7 is markedly overexpressed in tumors compared with normal tissues and growing studies have demonstrated the association of CXCR7 upregulation with tumor growth, neovascularization, invasion and metastasis [21, 22]. Activation of CXCL12/CXCR7 biased signal pathway may be critical for tumor progression by promoting cancer cell invasion and stem cell phenotype. It has been shown that nuclear YAP1 levels positively correlate with tumor grade, metastasis and induction of CSC-like activity [23]. However, whether CXCL12/CXCR7 biased signal contributes to CRC invasion and metastasis through YAP1 nuclear translocation and subsequent target gene regulation remains elusive. In this study, we unravel the intricate roles of CXCL12/CXCR7 biased signal on EMT and metastatic phenotype. More importantly, we reveal that YAP1 nuclear translocation activated by the biased signal plays a pivotal role in the regulation of EMT by repressing miRNAs. All these findings raise the exciting possibility that blocking CXCL12/CXCR7 biased signal pathway may be a valid strategy to inhibit metastasis of CRC.

## Results

### CXCR7 overexpression promotes EMT and upregulates the expression of stem marker DCLK1

**Fig. 1:**
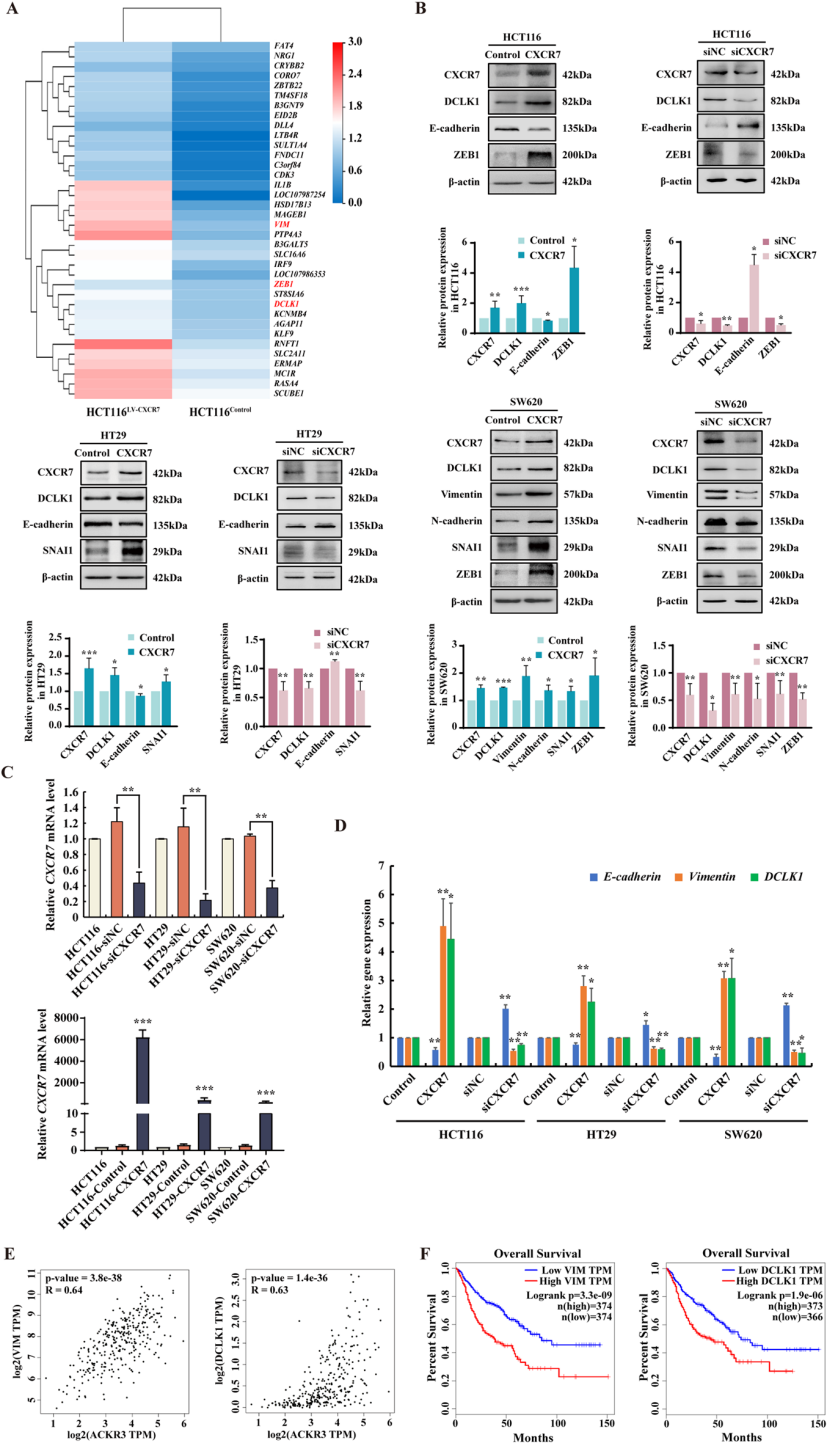
CXCR7 overexpression promotes EMT and upregulates the expression of DCLK1. **A** RNA-sequencing was performed in HCT116 cells infected with lentiviral expressed CXCR7 (LV-CXCR7) and control. Hierarchical clustering analysis of differentially expressing mRNAs between HCT116^Control^ and HCT116^LV−CXCR7^. **B** Western blot analysis of the expression levels of CXCR7, DCLK1, E-cadherin, Vimentin, N-cadherin, ZEB1 and SANI1 in HCT116, SW620 and HT29 cells infected with LV-CXCR7 and control lentivirus or transfected with siCXCR7 and siNC. Statistical analysis was performed compared with control group normalized to β-actin. Bars are means ± SD, *P < 0.05, **P < 0.01, ***P < 0.001, n = 3. **C**, **D** RT-qPCR was performed to determine the mRNA levels of *CXCR7*, *E-cadherin*, *Vimentin* and *DCLK1* in HCT116, HT29 and SW620 cells overexpressing or knockdown of CXCR7. Statistical analysis was performed compared with control group. Bars are means ± SD, *P < 0.05, **P < 0.01, ***P < 0.001, n = 3. **E** The correlation of CXCR7 (ACKR3) with Vimentin and DCLK1 was determined in CRC tissues by Gene Expression Profiling Interactive Analysis (GEPIA) online tools. **F** The overall survival analysis of gastrointestinal cancer patients (gastric adenocarcinoma plus colorectal adenocarcinoma) divided by expression of Vimentin and DCLK1 using GEPIA. The patients were divided with high and low gene expression levels using the median cutoff and log-rank *P* value was shown

CXCR7 is highly expressed in many cancers and has predominantly pro-metastatic roles in cancer [24]. EMT is a process by which polarized epithelial cells are transformed into mesenchymal cells with the properties of increased motility and invasion. It is characterized by decreasing of E-cadherin and increasing of Vimentin expression. To explore whether CXCR7 contributes to EMT, we performed RNA-sequencing in CXCR7-overexpressing HCT116 cells and control cells. Among all the differentially expressed genes, *Vimentin and ZEB1* were significantly increased and the intestinal stem cell marker *DCLK1* was also markedly enhanced when CXCR7 was overexpressed (Fig. 1A). The most significantly upregulated genes were listed in Additional file 2: Table S1. To further confirm the association of CXCR7 on the regulation of EMT and DCLK1, HCT116, HT29 and SW620 cells were infected by lentivirus expressing CXCR7 (LV-CXCR7) and siRNA targeting CXCR7 (siCXCR7). Firstly, we examined the basal levels of CXCR7 expression in these cells (Additional file 1: Fig. S1A, B), the results showed that HCT116 and HT29 cells have moderate expression levels of CXCR7 while SW620 cells have a higher expression level of CXCR7 than the other cells. HCT116 cells have the epithelial phenotype with high expression of E-cadherin but minimal level of mesenchymal marker Vimentin expression at protein levels. In contrast, SW620 cells have the high metastatic capacity to lymph node with high expression of Vimentin but trace amount of E-cadherin expression at protein levels. HT29 cells are more similar to HCT116 cells in phenotype and gene expression. Therefore, we initially selected the three cell lines for manipulation of CXCR7 expression. The results indicated that we can successfully overexpress and knockdown of CXCR7 in these cells (Fig. 1B, C). Particularly, there was no significant difference between parental cells and vector control or scramble siRNA in *CXCR7* expression (Fig. 1C). Interestingly, although there was genetic heterogeneity among the CRC cells, they displayed a consistent EMT phenotype when CXCR7 was overexpressed. Notably, Vimentin was prominently upregulated with concurrent downregulation of E-cadherin in CXCR7-overexpressing cells compared with vector control. In addition, EMT-related transcriptional factors ZEB1 and SNAI1 were also found to be upregulated when CXCR7 was overexpressed. Transwell assay indicated that the invasive capacity was potentially enhanced in CXCR7-overexpressing CRC cells compared with that of control cells (Additional file 1: Fig. S2). In contrast, these EMT-related proteins were downregulated when the cells were transfected with CXCR7 siRNA (Fig. 1B). RT-qPCR analysis further confirmed the regulation of *E-cadherin* and *Vimentin* at mRNA level by overexpressing or knockdown of CXCR7 (Fig. 1C, D). In parallel, as a stem marker associated with metastasis, DCLK1 had similar changes in line with EMT progression, suggesting overexpression of CXCR7 contributed to CRC metastasis by upregulating DCLK1 expression.

To determine the clinical relevance of CXCR7 expression with EMT, we analyzed the correlation of the expression of CXCR7 (ACKR3) with EMT markers and DCLK1 in human CRC tissues by Gene Expression Profiling Interactive Analysis (GEPIA) (http://GEPIA.cancer-pku.cn/index.html) using TCGA datasets. Notably, we found a robust and statistically significant association between Vimentin, DCLK1 and CXCR7 (R = 0.64 and 0.63 respectively, *P* < 0.001) (Fig. 1E). We also found a significant positive correlation between CXCR7 and EMT-related transcriptional factors (ZEB1 and SNAI1) respectively (Additional file 1: Fig. S3). These TCGA datasets analysis strongly support the association of the expression of CXCR7 with EMT. Furthermore, we evaluated the expression of Vimentin and DCLK1 as prognostic gene signature by using gastrointestinal cancer datasets, we found that high Vimentin and DCLK1 mRNA expression significantly correlated with a worse overall survival in gastrointestinal cancer (Fig. 1F). Collectively, these findings suggest that overexpression of CXCR7 promotes EMT and upregulates the expression of DCLK1, which is possibly associated with poor clinical outcome.

### CXCR7 overexpression contributes to EMT by repressing miR-124-3p and miR-188-5p

**Fig. 2:**
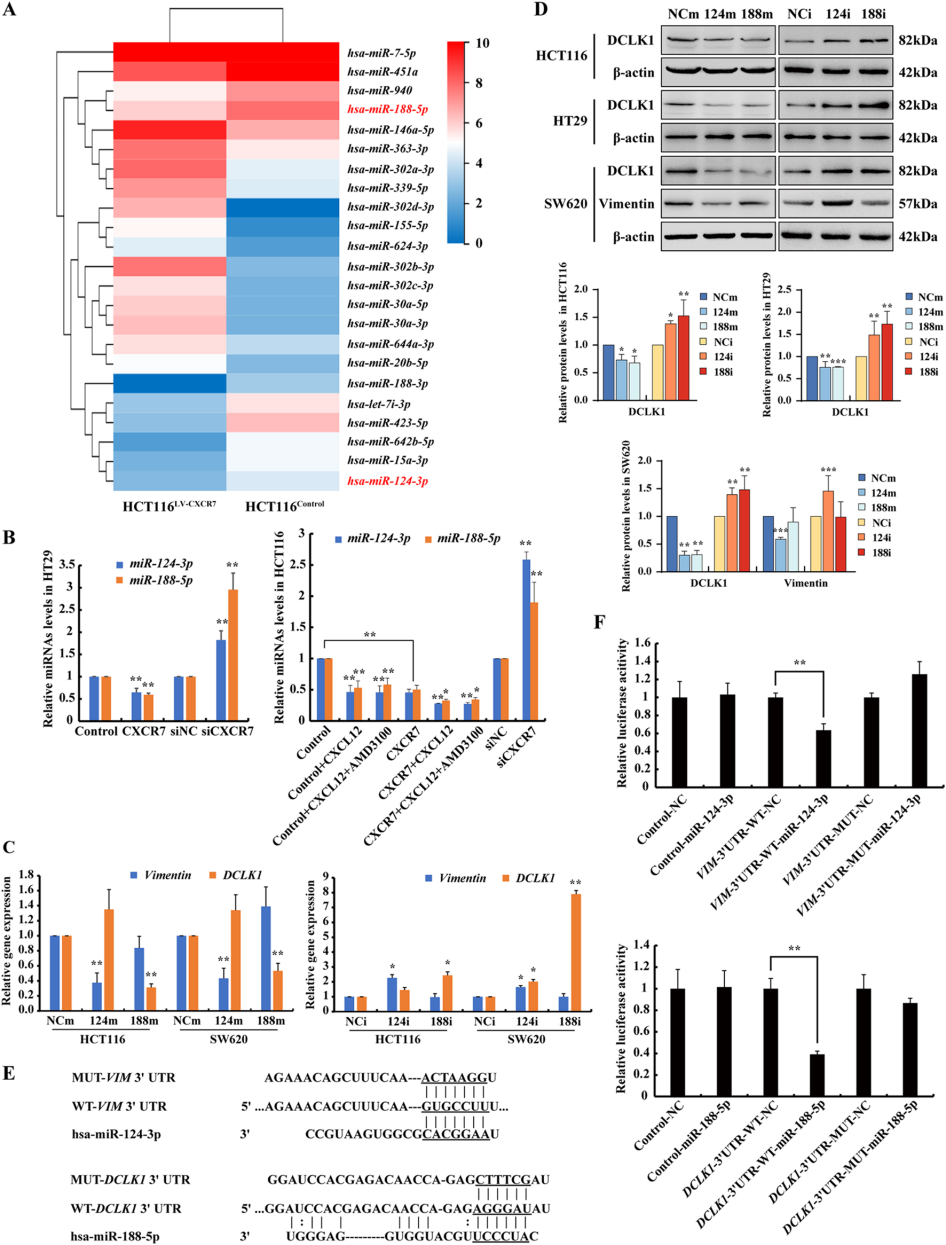
CXCR7 biased signal activation contributes to EMT by repressing miR-124-3p and miR-188-5p. **A** RNA-sequencing was performed in HCT116 cells infected with CXCR7 and control lentivirus. Hierarchical clustering analysis of differentially expressing miRNAs between HCT116^Control^ and HCT116^LV−CXCR7^ cells. **B** RT-qPCR analysis of *miR-124-3p* and *miR-188-5p* levels in HCT116 and HT29 cells infected with CXCR7 lentivirus or siRNA-CXCR7 and Controls with or without CXCL12 (100 ng/ml) in the presence of AMD3100 (2 μM) stimulation for 48 h. Statistical analysis was performed compared with vector control or siNC groups. *P < 0.05, **P < 0.01, n = 3 **C**, **D** RT-qPCR and Western blot analysis of DCLK1and Vimentin at mRNA and protein levels in CRC cells transfected with miR-124-3p and miR-188-5p mimics (124m, 188m) or inhibitors (124i, 188i) and their respective negative control (NCm, NCi). β-actin was used as loading control. Statistical analysis was performed compared with control group. Bars are means ± SD, *P < 0.05, **P < 0.01, ***P < 0.001, n = 3. **E**, **F** HCT116 cells were co-transfected with DCLK1 and Vimentin luciferase constructs and negative control (NC), miR-188-5p and miR-124-3p mimics respectively. The comparison of luciferase activity of wild-type (WT) and mutant (MUT) *DCLK1*-3′UTR or *Vimentin*-3′UTR constructs was performed 36 h after transfection. Con081 luciferase plasmid was used as the vector control. Data was normalized to Renilla activity. Bars are means ± SD; *P < 0.05, **P < 0.01, n = 3

In order to investigate the mechanism that CXCR7 signal activation contributes to CRC progression and EMT, miRNA sequencing was performed in above HCT116 cells (HCT116^Control^ and HCT116^LV−CXCR7^), and the significantly upregulated and downregulated miRNAs were listed in Additional file 2: Table S2. Among all the differentially expressed miRNAs, *miR-124-3p* and *miR-188-5p* were significantly downregulated in HCT116^LV−CXCR7^ compared with HCT116^Control^, indicating that these miRNAs were downregulated by CXCR7 activation (Fig. 2A). To verify the results, we determined the expression of these miRNAs in CRC cells in response to activation of the CXCL12/CXCR7 axis. As a result, RT-qPCR analysis showed that *miR-124-3p* and *miR-188-5p* were significantly downregulated by overexpression of CXCR7, which was further suppressed in response to CXCL12 stimulation. In contrast, knockdown of CXCR7 markedly elevated these miRNAs in HCT116 and HT29 cells (Fig. 2B). CXCL12 is known as the common ligand for activation of CXCR4 and CXCR7, to elucidate whether activation of CXCR7 represses the expression of miRNAs through biased signaling, we used AMD3100, the specific inhibitor of CXCR4, to exclude the effect of activation of CXCL12/CXCR4 axis. The results showed that CXCL12/CXCR7 produced a similar effect on the downregulation of *miR-124-3p* and *miR-188-5p* with or without the treatment of AMD3100. Specifically, the biased signal of CXCL12/CXCR7 profoundly suppressed *miR-124-3p* and *miR-188-5p*. (Fig. 2B).

To gain an insight into the molecular mechanism of these miRNAs on CRC progression and EMT, two mRNA target-predicting algorithms (MiRDB and Targetscan) were utilized to identify the potential downstream targets of miR-124-3p and miR-188-5p. *Vimentin and DCLK1* were predicted to be the potential target gene of miR-124-3p and miR-188-5p respectively. To verify the hypothesis, HCT116, HT29 and SW620 cells were transfected with miR-124-3p and miR-188-5p mimics and inhibitors respectively, and the results showed that these miRNAs were significantly enhanced or suppressed in these CRC cells (Fig. S4A, B). As expected, the expression of Vimentin was significantly suppressed by miR-124-3p mimics, while substantially enhanced by miR-124-3p inhibitors. Similarly, the expression of DCLK1 was robustly reduced by miR-188-5p mimics and increased by miR-188-5p inhibitors (Fig. 2C, D). Of note, we also observed the regulation of DCLK1 by miR-124-3p, possibly due to the indirect regulatory effect of miR-124-3p on DCLK1 gene expression since miR-124-3p has multiple target genes and exhibits tumor suppressive effect in colorectal cancer [25]. Furthermore, luciferase reporter assay was performed to confirm the direct binding of miR-124-3p with *Vimentin* 3′-UTR and miR-188-5p with *DCLK1* 3′-UTR respectively. The predicted binding sites of the miRNAs with wild type and mutant 3′-UTR luciferase reporter constructs are shown in Fig. 2E. In accordance with these results, the binding was abolished by mutation of the binding sites of these miRNAs on *Vimentin* 3′-UTR and *DCLK1* 3′-UTR, suggesting that these miRNAs could directly bind to *Vimentin* 3′-UTR and *DCLK1* 3′-UTR and regulate its expression at the post-transcriptional level (Fig. 2F). Taken together, these results establish that CXCR7 biased signal activation contributes to CRC progression and EMT by repressing miR-124-3p and miR-188-5p that targeting *Vimentin* and *DCLK1*.

### YAP1 manipulates CXCR7 biased activation-induced EMT by repressing miR-124-3p and miR-188-5p

EMT is a highly dynamic and reversible process, conferring cancer cells with the plasticity for distant dissemination and invasive capacity. YAP1/TEAD transcriptional activation has emerged as the important regulator of EMT by cooperation with ZEB1 and AP-1 [26]. Based on this, we hypothesize that YAP1 promotes EMT via the regulation of miRNAs in CRC cells.

**Fig. 3:**
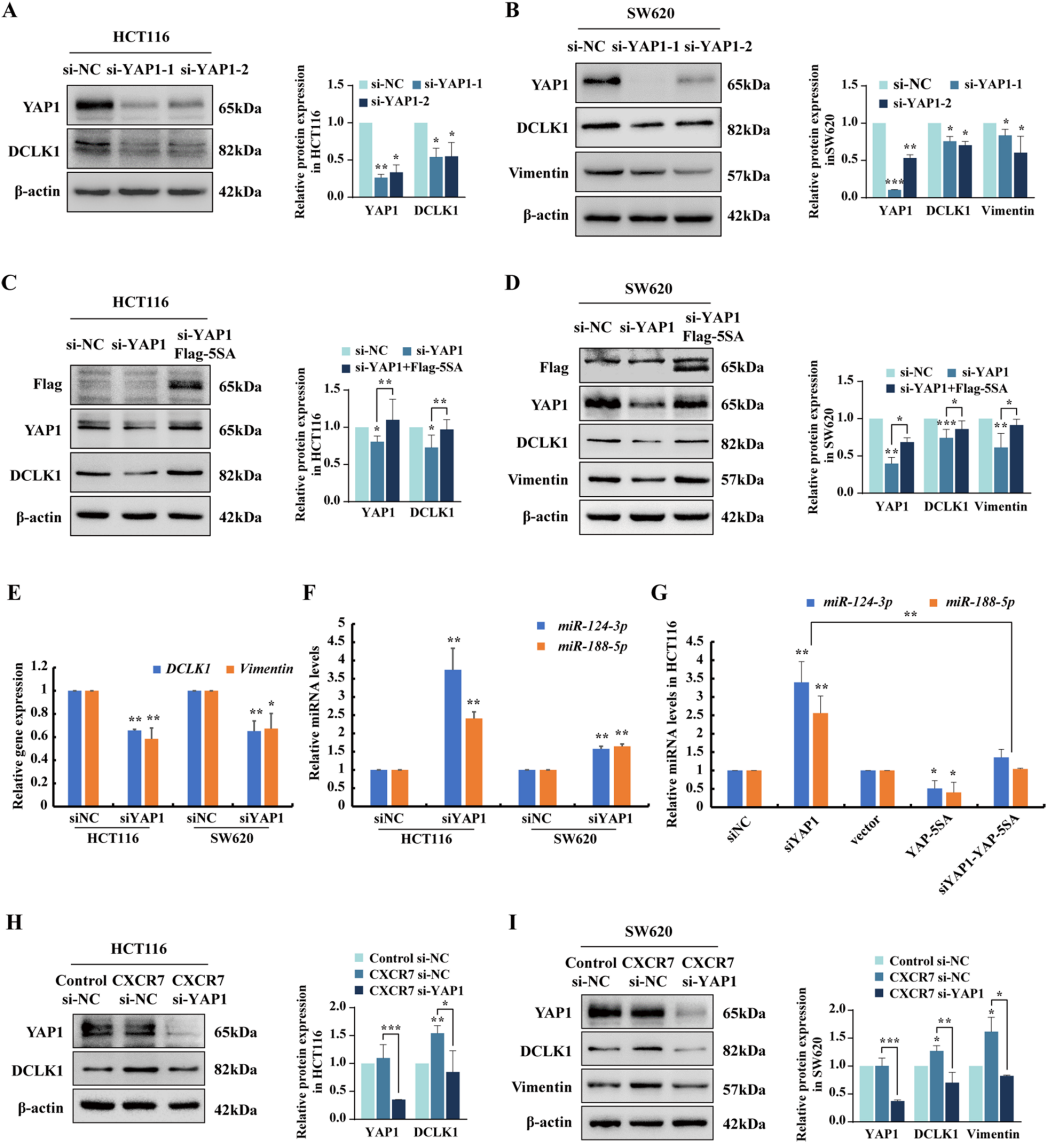
YAP1 promotes EMT and upregulates DCLK1 by repressing miR-124-3p and miR-188-5p. **A**, **B** Western blot analysis of the expression levels of YAP1, DCLK1 or Vimentin in HCT116 and SW620 cells transfected with two different YAP1 siRNAs. β-actin was used as loading control. Statistical analysis was performed compared with siNC group. **C**, **D** Western blot analysis of YAP1, DCLK1 or Vimentin in HCT116 and SW620 cells transfected with YAP1 siRNA and then rescued with transfection of Flag-YAP-5SA plasmid. Flag tag was used to indicate the overexpression of YAP-5SA. **E**, **F** RT-qPCR analysis of *DCLK1* and *Vimentin* at mRNA levels and concurrent expression levels of miR-124-3p and miR-188-5p in HCT116 and SW620 cells transfected with YAP1 siRNAs. Statistical analysis was performed compared with siNC group. **G** RT-qPCR analysis of expression levels of *miR-124-3p* and *miR-188-5p* in HCT116 cells transfected with YAP1 siRNA and Flag-YAP-5SA plasmid compared with siNC and vector control respectively. **H**, **I** Western blot analysis of YAP1, DCLK1 or Vimentin in CXCR7-overexpressing HCT116 and SW620 cells transfected with YAP1 siRNA and siNC. The vector control CRC cells were transfected with siNC as controls. Bars are means ± SD; *P < 0.05, **P < 0.01, ***P < 0.001 (n = 3)

Knockdown of YAP1 with two different siRNAs led to a pronounced decrease of DCLK1 and Vimentin at protein levels in CRC cells (Fig. 3A, B). As a transcriptional coactivator, YAP1 translocates into nucleus to exert the regulatory effects with TEAD that contains an N-terminal DNA-binding domain. To further investigate whether nuclear YAP1 orchestrates the expression of Vimentin and DCLK1 by regulating miRNAs expression, we transfected CRC cells with a construct expressing the constitutive active form of YAP1 (YAP-5SA), resistant to LATS-mediated phosphorylation, which directly leads to YAP1 nuclear translocation. Intriguingly, enforced expression of YAP-5SA rescued, to a large extent, the marked downregulation of DCLK1 and Vimentin caused by YAP1 knockdown (Fig. 3C, D). Moreover, overexpression of Flag-YAP-5SA significantly enhanced the expression of DCLK1 and Vimentin compared with vector control in both HCT116 and SW620 cells (Additional file 1: Fig. S5). Interestingly, RT-qPCR analysis further confirmed the downregulation of mRNA levels of *DCLK1* and mesenchymal marker *Vimentin* upon YAP1 knockdown. Of relevance, the expression levels of *miR-124-3p* and *miR-188-5p* were strongly enhanced by YAP1 silencing (Fig. 3E, F).

More importantly, enforced expression of YAP-5SA resulted in a drastic reduction of *miR-124-3p* and *miR-188-5p* and it could also attenuate the prominent elevation of these miRNAs in YAP1 knockdown cells (Fig. 3G). These results suggested that YAP-5SA rescued the suppression of EMT markers caused by silencing of YAP1 through the repression of miR-124-3p and miR-188-5p. To further prove that YAP1 is involved in the regulation of CXCR7-induced EMT, CRC cells overexpressing CXCR7 were transfected with siYAP1, as a result, knockdown of YAP1 significantly suppressed the upregulation of Vimentin and DCLK1 induced by overexpression of CXCR7 (Fig. 3H, I). Taken together, these findings reveal that nuclear YAP1 critically promoted CXCR7-induced EMT by repressing miR-124-3p and miR-188-5p in CRC cells.

### CXCR7/β-arr1-mediated biased signal induces YAP1 nuclear translocation in CRC cells

**Fig. 4:**
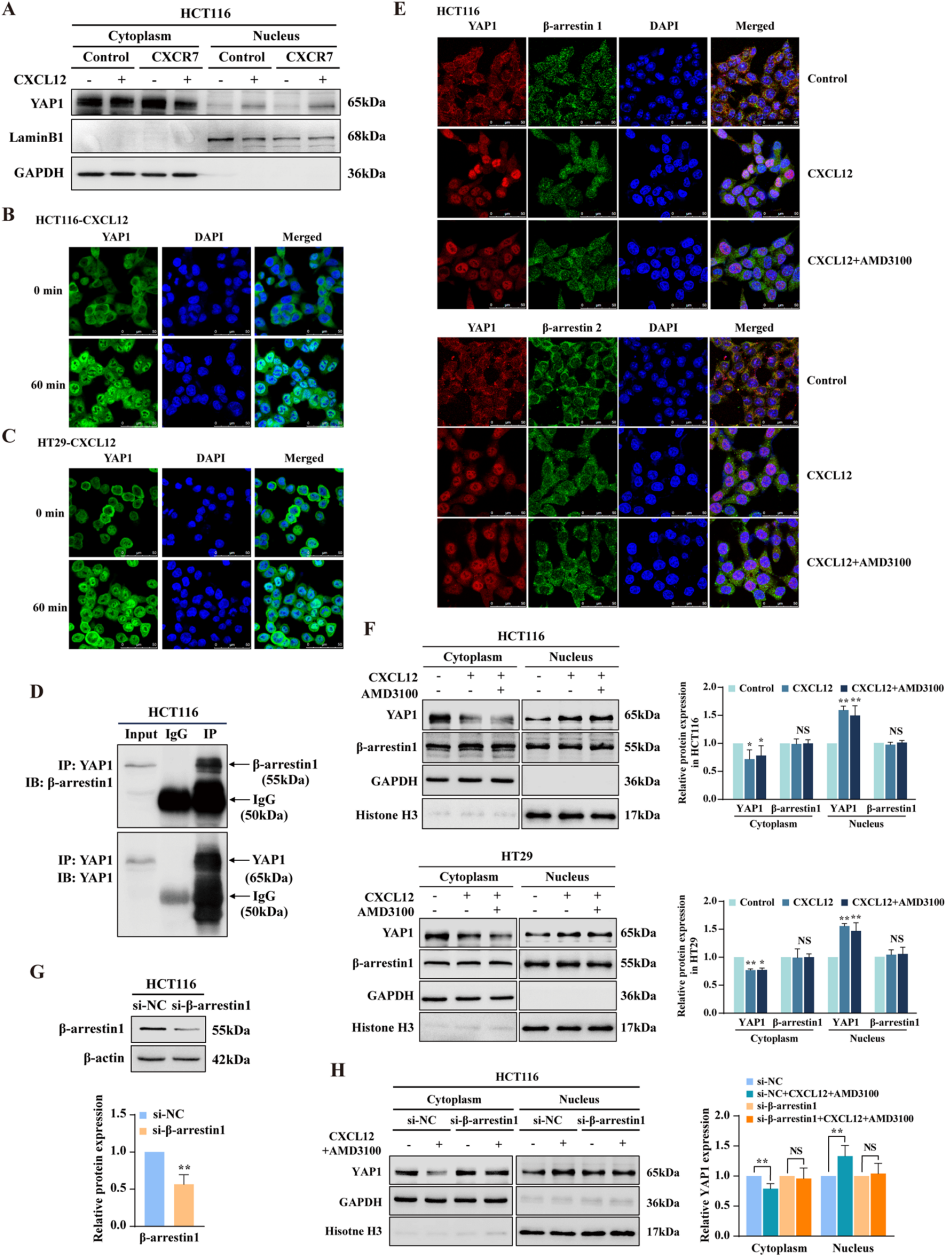
CXCR7/β-arr1-mediated biased signal induces YAP1 nuclear translocation in CRC cells. **A** Western blot analysis of YAP1 expression in cytoplasmic and nuclear extracts of HCT116^Control^ and HCT116^LV−CXCR7^ cells treated with or without CXCL12 (100 ng/ml) for 60 min. GAPDH and Lamin B1 were used as cytoplasmic and nuclear loading control, respectively. **B**, **C** YAP1 localization evaluated by immunofluorescence (IF) in HCT116 and HT29 cells treated with or without CXCL12 (100 ng/ml). YAP1 was labeled with Alexa Fluor® 488 donkey anti-rabbit secondary antibodies, nuclei were visualized with DAPI, shown in blue. Scale bars, 50 µm. **D** Analysis of endogenous YAP1-β-arr1 interaction in HCT116 cells by Co-immunoprecipitation (Co-IP). Normal rabbit IgG antibodies were used as control. **E** IF staining was performed to determine the colocalization of YAP1 (red) and β-arrestin1 (green) or β-arrestin2 (green) in HCT116 cells treated with CXCL12 (100 ng/ml) in the presence of AMD3100 (2 μM). DAPI was used for nuclear staining. Scale bars, 50 µm. **F** Western blot analysis of YAP1 and β-arrestin1 expression in cytoplasmic and nuclear extracts of HCT116 and HT29 cells treated with or without CXCL12 (100 ng/ml) in the presence of AMD3100 (2 μM). GAPDH and Histone H3 were used as cytoplasmic and nuclear loading control, respectively. **G** Western blot analysis of β-arrestin1 expression in HCT116 cells transfected with β-arrestin1 siRNA. β-actin was used as loading control. **H** Western blot analysis of YAP1 expression in cytoplasmic and nuclear extracts of HCT116 cells transfected with β-arrestin1 siRNA or siNC and treated with or without CXCL12 (100 ng/ml) plus AMD3100 (2 μM). GAPDH and Histone H3 were used as cytoplasmic and nuclear loading control, respectively. Bars are means ± SD; *P < 0.05, **P < 0.01, ***P < 0.001, NS stands for no significance (n = 3)

Since nuclear YAP1 plays a crucial role in regulation of EMT by repression of miR-124-3p and miR-188-5p, next, we explored whether CXCL12/CXCR7 biased activation promotes YAP1 nuclear translocation. Notably, CXCL12 induced the reduction of YAP1 in the cytoplasm paralleled with YAP1 nuclear accumulation, which was potentiated by overexpression of CXCR7 (Fig. 4A). The CXCL12-induced YAP1 nuclear accumulation was also confirmed by immunofluorescence analysis in HCT116 and HT29 cells, showing the time-dependent nuclear translocation of YAP1 and reached plateau after 60 min stimulation by CXCL12 (Fig. 4B, C and Additional file 1: Fig. S6).

β-arr1, previously known as a cytosolic regulator and scaffold of GPCR signaling, has recently been revealed to translocate to the nucleus mediating receptor endocytosis and signal transduction. Therefore, it is likely that β-arr1 shuttles between the cytoplasm and the nucleus mediating CXCL12/CXCR7 biased signal transduction. To assess whether β-arr1 could functionally contribute to YAP1 activity regulation consequently to CXCL12/CXCR7 axis activation, we performed co-immunoprecipitation analysis in whole cell lysates derived from HCT116 cells, and found that endogenous YAP1 could physically interact with β-arr1 (Fig. 4D). Here we ask whether the interaction between β-arr1 and YAP1 facilitates the nuclear translocation of YAP1 with the concurrent nuclear shuttling of β-arr1 or not? Immunofluorescence analysis indicated that although CXCL12 stimulation at early stage (for 90 min) led to substantial YAP1 nuclear translocation, β-arr1 and β-arr2 were predominantly located in the cytoplasm and not involved in this process (Fig. 4E). However, it was true that we observed the nuclear translocation of β-arr1 at later stage (> 6 h) of CXCL12 stimulation (Additional file 1: Fig. S7). Furthermore, nuclear-cytoplasmic fractions indicated that CXCL12 induced dramatic reduction of YAP1 in the cytoplasm accompanied by the significant increase of nuclear YAP1. Concurrently, AMD3100, as a CXCR4 antagonist, was used to rule out any effects of CXCL12/CXCR4 signaling activation (Fig. 4F). These results indicated that CXCL12/CXCR7 biased signal activation substantially promoted nuclear translocation of YAP1. Noticeably, β-arr1 did not exhibit a simultaneous nuclear translocation at early stage of CXCL12 stimulation in CRC cells (Fig. 4F). Then we ask whether β-arr1 is required for YAP1 nuclear translocation upon CXCL12/CXCR7 biased signal activation? As shown in Fig. 4G, H, knockdown of β-arr1 interfered with the nuclear translocation of YAP1 in response to CXCL12 stimulation.

These results reveal that CXCL12/CXCR7 biased signal activation promotes YAP1 nuclear translocation by recruiting β-arr1 in the cytoplasm, which could not be abrogated by AMD3100 pretreatment. Overall, these findings establish that CXCR7 activation by CXCL12 induces YAP1 nuclear enrichment in CRC cells and prove the critical role of β-arr1 in transducing CXCL12/CXCR7-dependent YAP1 cytoplasmic-nuclear shuttling.

### YAP1 inhibits miR-124-3p and miR-188-5p expression by recruiting YY1 to the promoters

Nuclear YAP1 functions as a potent transcriptional cofactor by binding with TEAD1. YAP1/TEAD1 complex interacts with other transcriptional factors to regulate the expression of target genes [27]. Generally, YAP1/TEAD complex activates the oncogenic downstream genes to trigger carcinogenesis. To further explore the inhibitory effects of nuclear YAP1 on the expression of miR-124-3p and miR-188-5p, TransmiR v2.0 database (http://www.cuilab.cn/transmir) and interface of mirTrans (http://mcube.nju.edu.cn/jwang/lab/soft/mirtrans/) were used to predict the transcriptional factor binding sites at the promoters of *miR-124-3p* and *miR-188-5p*. As a result, Yin Yang 1 (YY1) was predicted to be the potentially common transcriptional factor that could suppress the expression of these miRNAs (Additional file 1: Fig. S8).

**Fig. 5:**
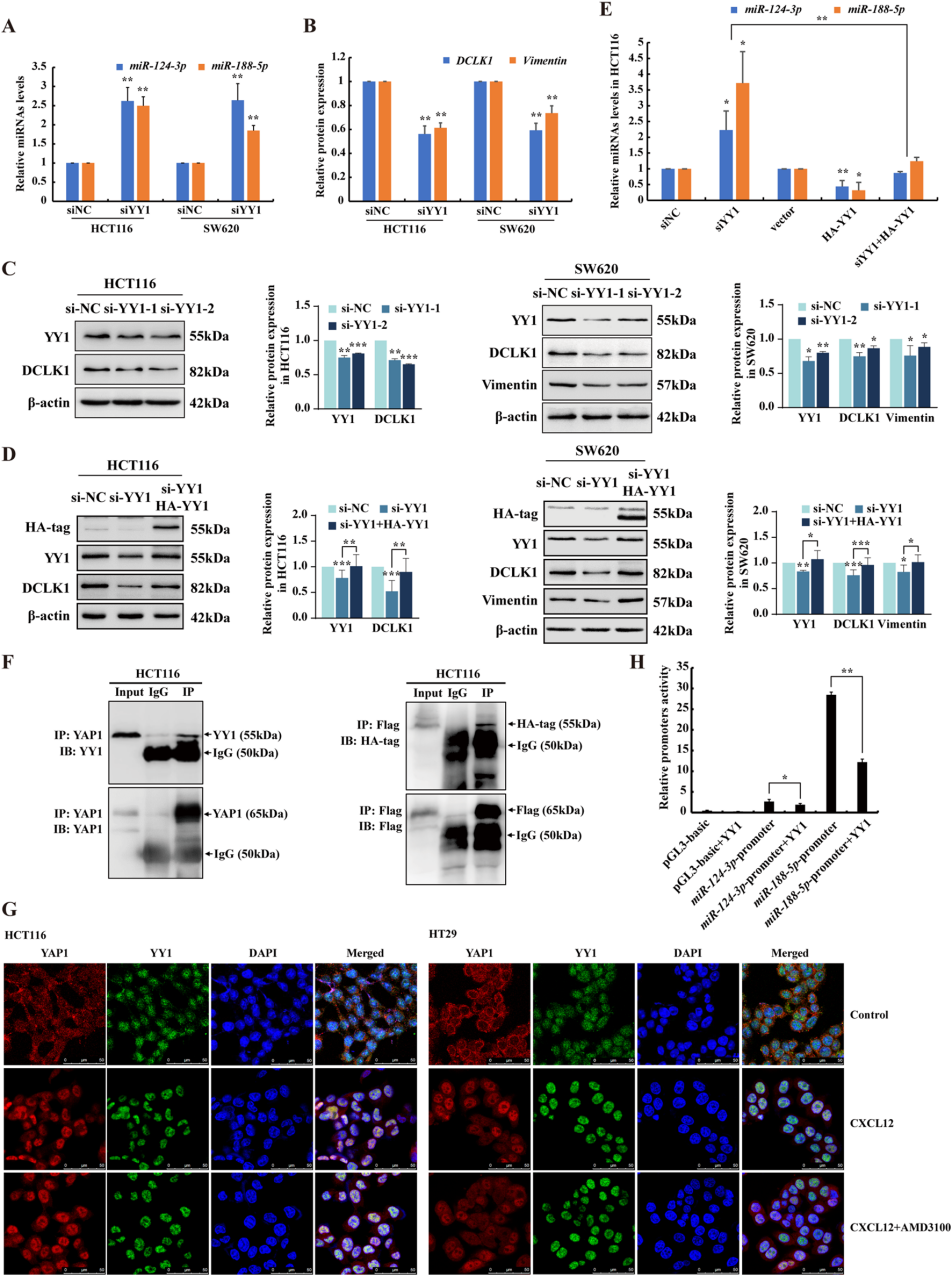
YAP1 inhibits miR-124-3p and miR-188-5p expression by recruiting YY1 to the promoters. **A**, **B** RT-qPCR analysis of the mRNA levels of *DCLK1*, *Vimentin* and concurrent expression levels of *miR-124-3p* and *miR-188-5p* in HCT116 and SW620 cells transfected with YY1 siRNAs. **C** Western blot analysis of the expression of YY1, DCLK1 or Vimentin in HCT116 and SW620 cells transfected with two different YY1 siRNAs. β-actin was used as an internal control. **D** Western blot analysis of YY1, DCLK1 or Vimentin in HCT116 and SW620 cells transfected with YY1 siRNA and then rescued with HA-YY1 plasmid. **E** RT-qPCR analysis of *miR-124-3p* and *miR-188-5p* levels in HCT116 cells transfected with YY1 siRNA and HA-YY1 plasmid compared with siNC and vector control respectively. Bars are means ± SD; *P < 0.05, **P < 0.01, ***P < 0.001, n = 3. **F** Analysis of the interaction of endogenous YAP1 with YY1 and transfected Flag-YAP-5SA with HA-YY1 by Co-IP. IP was performed using anti-YAP1 or anti-Flag antibodies and normal rabbit IgG antibodies. **G** IF staining was performed to determine the colocalization of YAP1 (red) and YY1 (green) in HCT116 and HT29 cells treated with CXCL12 (100 ng/ml) in the presence of AMD3100 (2 μM). DAPI was used for nuclear staining. Scale bars, 50 µm. (**H**) HCT116 cells were co-transfected with *miR-124-3p* or *miR-188-5p* promoter luciferase construct (*miR-124* or *miR-188* promoter) together with HA-YY1 plasmid. pGL3-basic plasmid was used as the vector control. The comparison of luciferase activities of promoter constructs normalized to Renilla activity was indicated. Bars are means ± SD; *P < 0.05, **P < 0.01 (n = 3)

We hypothesized that YAP1 functions as a transcriptional repressor by interacting with YY1, transcriptionally repressing the expression of miR-124-3p and miR-188-5p, which promotes EMT and metastasis. To prove this hypothesis, HCT116 and SW620 cells were used to confirm whether YY1 is involved in the regulation of miR-124-3p and miR-188-5p expression. As shown in Fig. 5A, *miR-124-3p* and *miR-188-5p* were robustly upregulated by YY1 silencing. Expectedly, knockdown of YY1 led to remarkably downregulation of mesenchymal marker Vimentin in SW620 cells. Consistently, the expression of DCLK1 was also profoundly impaired with YY1 depletion in both HCT116 and SW620 cells (Fig. 5B, C). These results indicated that YY1 was implicated with the regulation of EMT. To further assess the specificity of YY1 in promoting EMT, we transfected CRC cells with a construct expressing YY1 with HA-tag which led to YY1 overexpression. Remarkably, YY1 silencing led to a marked reduction of the expression of Vimentin and DCLK1, which could be rescued by enforced expression of HA-YY1 at protein levels (Fig. 5D). In particular, overexpression of YY1 could enhance the expression of DCLK1 and Vimentin in CRC cells (Additional file 1: Fig. S9). In order to prove that YY1 is required for promoting EMT by repressing the expression levels of miR-124-3p and miR-188-5p, we performed RT-qPCR analysis and found that YY1 silencing significantly enhanced the levels of *miR-124-3p* and *miR-188-5p*, which can be strongly attenuated by enforced expression of HA-YY1 (Fig. 5E).

In order to address our hypothesis that YAP1 inhibits the expression of miR-124-3p and miR-188-5p by recruiting YY1, Co-IP assay was performed in HCT116 cells. We found that endogenous YAP1 could physically interact with YY1. Furthermore, when HCT116 cells were transfected with Flag-YAP-5SA and HA-YY1, YAP1 was found to interact with HA-YY1 in the nucleus by exogenously overexpression (Fig. 5F). Consistently, immunofluorescence analysis further showed the nuclear colocalization of YAP1 and YY1 in CRC cells upon CXCL12 stimulation with or without the treatment of AMD3100 (Fig. 5G). Moreover, luciferase reporter assay showed that the promoter activities of *miR-124-3p* and *miR-188-5p* were robustly enhanced compared with control vector of pGL3-basic, which was significantly impaired upon YY1 overexpression (Fig. 5H). Taken together, these results unveil that YAP1 inhibited miR-124-3p and miR-188-5p expression by recruiting YY1 to the promoters, therefore, they cooperated to regulate EMT plasticity and metastasis of CRC cells.

### YAP1 inhibitor suppresses CXCL12/CXCR7-induced EMT and tumor metastasis

**Fig. 6:**
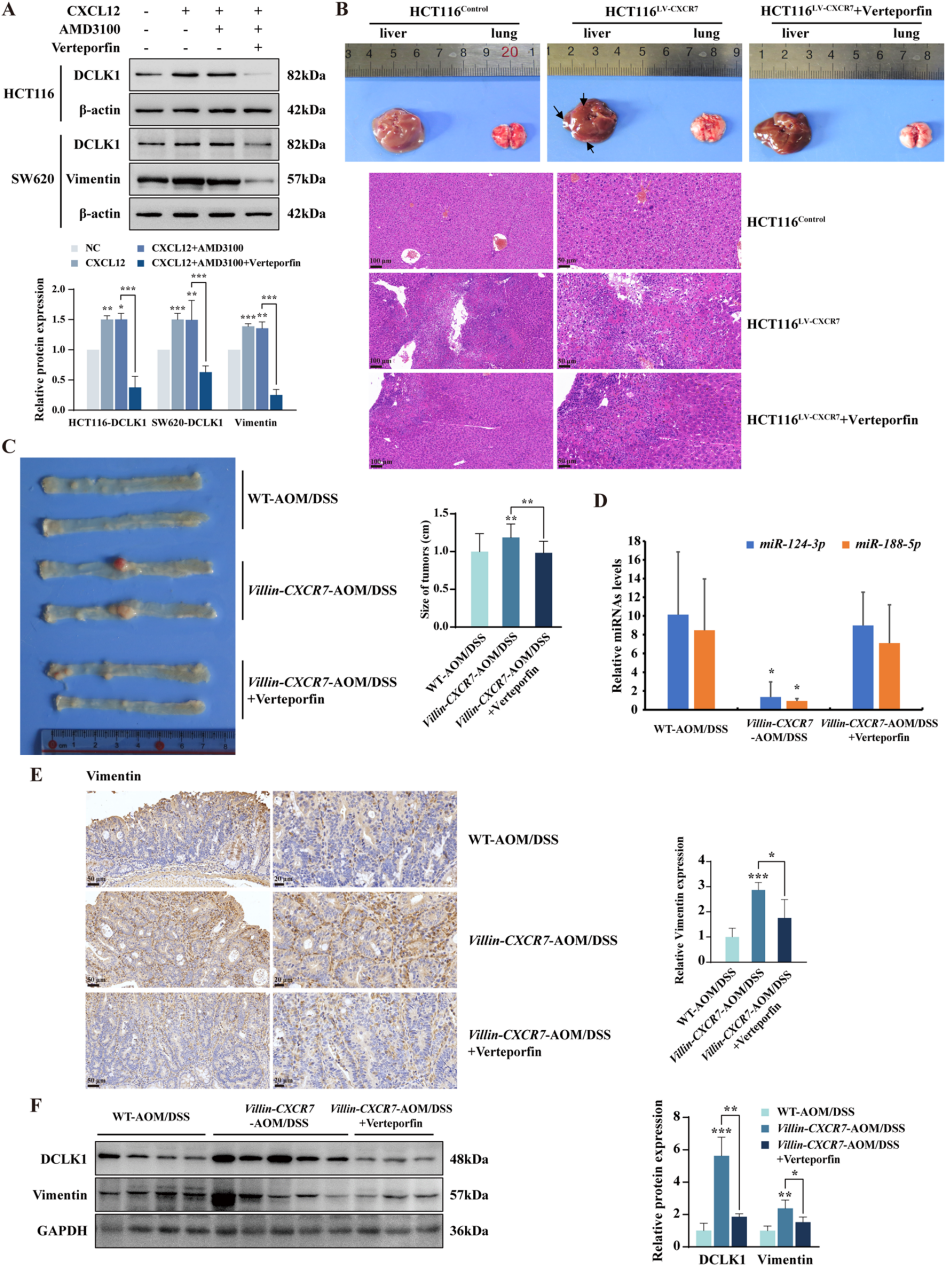
YAP1 inhibitor suppresses CXCR7 induced tumor progression and metastasis. **A** Western blot analysis of the expression of DCLK1 or Vimentin normalized to β-actin in HCT116 and SW620 cells treated with CXCL12 (100 ng/ml) in the presence of AMD3100 (2 μM) and Verteporfin (3 µM) for 48 h. **B** Representative images of livers and lungs and H&E-stained sections of liver metastatic nodules in nude mice inoculated with HCT116^LV−CXCR7^ and HCT116^Control^ cells via tail veins with or without the treatment of Verteporfin (10 mg/kg, n = 3 per group). The arrows point out the visible metastatic nodules of liver. **C** Representative images of colons from AOM/DSS-treated WT, *Villin-CXCR7* mice and *Villin-CXCR7* mice treated with Verteporfin (10 mg/kg). Average size of colon polyps was analyzed in different groups (n = 5). **D** RT-qPCR analysis of expression levels of *miR-124-3p* and *miR-188-5p* in colon cancer tissues from above-mentioned C57BL/6 mice. **E** Representative IHC staining of Vimentin in AOM/DSS-induced colon adenocarcinoma tissues from wild type C57BL/6 mice and *Villin-CXCR7* mice administered with Verteporfin (10 mg/kg) or vehicle control via intraperitoneal injection daily. **F** Western blot analysis of DCLK1 and Vimentin expression in colon cancer tissues from these mice. GAPDH was used as loading control and statistical analysis was performed. *P < 0.05, **P < 0.01, ***P < 0.001

In light of the crucial role of YAP1 in promoting EMT by repressing miR-124-3p and miR-188-5p via interacting with YY1, we wonder if YAP1 inhibitor Verteporfin could blunt CXCL12/CXCR7-induced EMT and distant metastasis. As shown in Fig. 6A, CXCL12/CXCR7 biased activation strongly upregulated the expression of DCLK1 and Vimentin, which was attenuated by Verteporfin. We further examined the effects of Verteporfin on distant metastasis when nude mice were injected with HCT116^LV−CXCR7^ cells via tail veins. The results indicated that CXCR7 overexpression facilitated more distant liver metastasis as shown by the larger metastatic nodules, which was greatly impaired by Verteporfin. The representative Hematoxylin and Eosin (HE) staining of metastatic nodules in livers were illustrated in Fig. 6B. However, there was no significant difference in lung metastasis among these groups.

To investigate the role of YAP1 in colitis-associated carcinogenesis and progression upon CXCL12/CXCR7 biased activation in vivo, wild type (WT) and *Villin-CXCR7* transgenic mice (*Villin-CXCR7*) were treated with AOM and DSS for 3 cycles as described in methods. We found that AOM/DSS treatment seriously aggravated colonic inflammation and tumor burden in *Villin-CXCR7* mice compared with WT mice, as indicated by the larger size of colonic adenocarcinoma. Importantly, Verteporfin, a YAP1 inhibitor which disrupts YAP-TEAD interactions, led to a significant suppression of the colonic adenocarcinomas (Fig. 6C). To investigate whether YAP1 inhibitor hinders EMT process in CRC by regulation of miR-124-3p and miR-188-5p in vivo, we performed RT-qPCR analysis and found that pharmacological inactivation of YAP1 with Verteporfin reversed the repression of miRNAs in AOM/DSS induced *Villin-CXCR7* mice (Fig. 6D). Further IHC and Western blot analysis showed that Vimentin and DCLK1 were highly expressed in colonic adenocarcinoma of *Villin-CXCR7* mice, which was abrogated by Verteporfin (Fig. 6E, F). These results suggested that YAP1 inhibitor significantly suppressed distant metastasis of HCT116^LV−CXCR7^ tumor xenografts in nude mice and AOM/DSS-induced colonic adenocarcinoma in *Villin-CXCR7* transgenic mice, highlighting the therapeutic potential of targeting YAP1 in the control of CRC progression and metastasis.

### CXCL12/CXCR7/β-arr1-induced YAP1 nuclear translocation is associated with EMT and metastasis in human CRC tissues

**Fig. 7:**
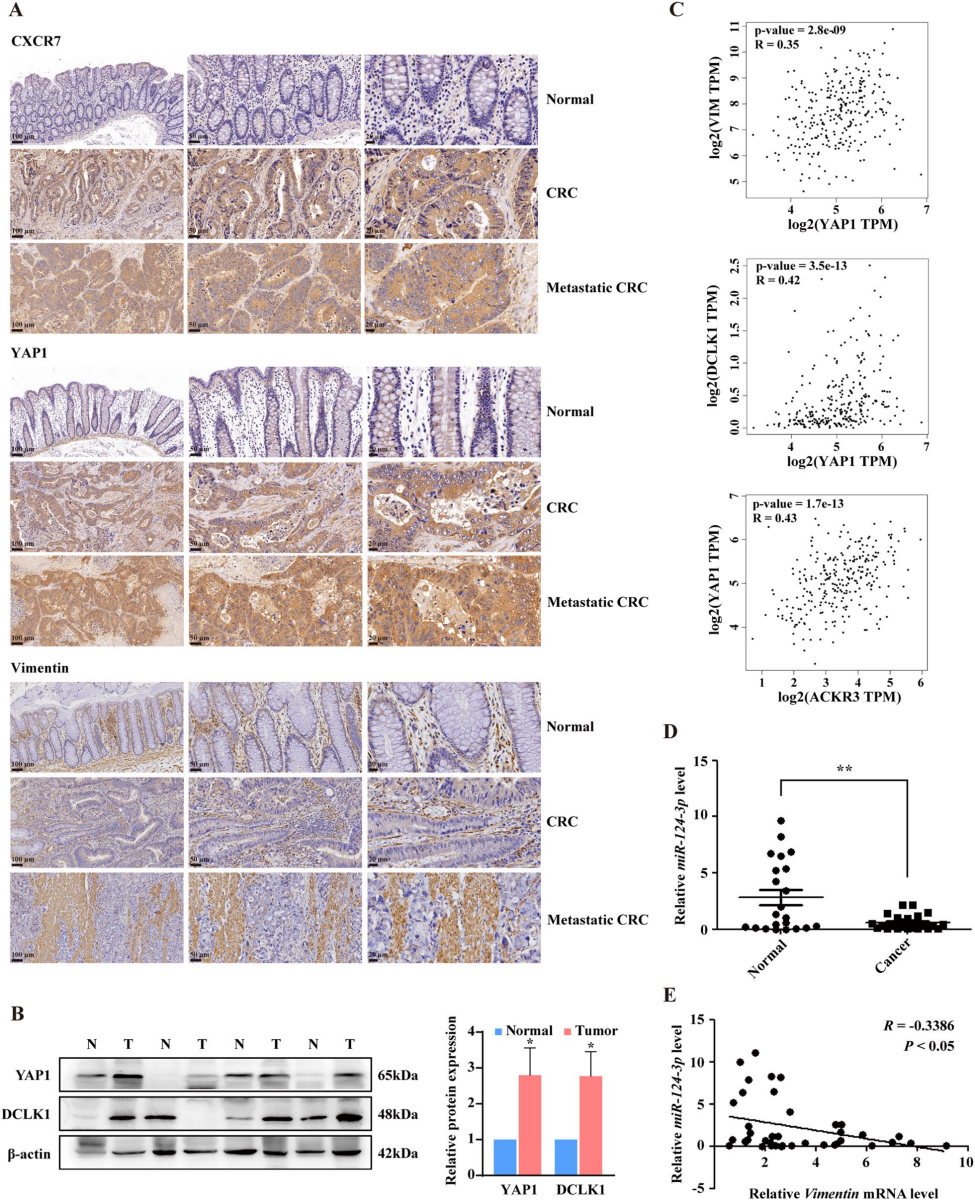
YAP1 nuclear translocation is associated with EMT and metastasis in human CRC tissues. **A** Representative immunohistochemistry images of the expression of CXCR7, YAP1 and Vimentin in paired normal colonic tissues, CRC tissues and matched liver metastatic CRC tissues from CRC patients. Scale bar = 100, 50, 20 μm. **B** Western blot analysis of YAP1 and DCLK1 expression in 22 pairs of human CRC tissues and adjacent normal tissues. β-actin was used as an internal control. Statistical analysis was performed to compare the gene expression in colorectal cancer tissues (T) compared with adjacent normal colonic tissues (N). *P < 0.05. **C** The correlations of YAP1 with DCLK1 and Vimentin as well as association of CXCR7 with YAP1 were determined in CRC tissues by GEPIA. **D** RT-qPCR analysis of *miR-124-3p* levels in 22 pairs of human CRC tissues and adjacent normal tissues. **E** The correlation of *miR-124-3p* and *Vimentin* mRNA level was performed by Pearson correlation analysis (n = 40). *P < 0.05, **P < 0.01

**Fig. 8:**
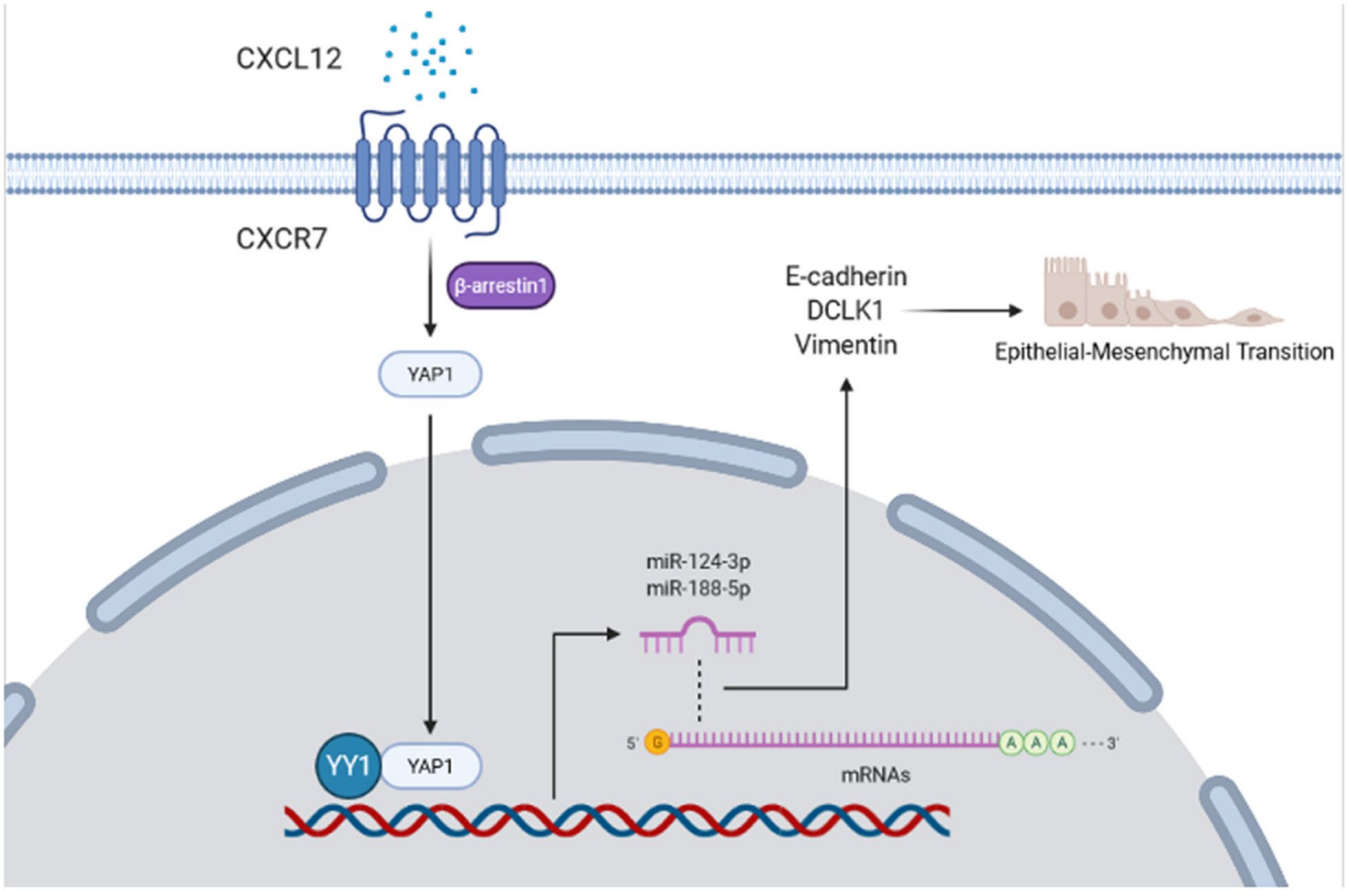
Schematic depiction of the working model for CXCL12/CXCR7/β-arrestin1 biased signal promoting EMT through YAP1 nuclear translocation

To explore the clinical relevance of CXCR7 activation with expression of nuclear YAP1 and EMT markers in colorectal cancer patients, we collected 22 pairs of human CRC specimens and adjacent normal colonic tissues for immunohistochemistry, Western blot and RT-qPCR analysis. As shown in Fig. 7A, CXCR7 was highly expressed in human CRC tissues compared with normal colon tissues, particularly with higher expression in metastatic CRC tissues than non-metastatic counterparts. YAP1 was remarkably overexpressed in nuclei in CRC tissues whereas predominantly expressed in cytoplasm in adjacent normal colon tissues. More importantly, there was also a higher expression of nuclear YAP1 in metastatic CRC tissues than non-metastatic counterparts. Similarly, the expression of Vimentin was higher in CRC particularly metastatic CRC tissues compared with adjacent normal colon tissues (Fig. 7A). Further protein analysis exhibited prominently high expression of YAP1 and DCLK1 in CRC tissues compared with adjacent normal tissues (Fig. 7B). To explore the potential link between YAP1, Vimentin, DCLK1 and upstream CXCR7, we used public accessible online tool GEPIA (http://GEPIA.cancer-pku.cn/index.html). It revealed a significant positive correlation of the expression of YAP1 and Vimentin (R = 0.35, *P* < 0.001) as well as other EMT-related transcriptional factors (Additional file 1: Fig. S10). In addition, the expression of YAP1 and DCLK1 also displayed a potent correlation (R = 0.42, *P* < 0.001) (Fig. 7C). Notably, the expression level of CXCR7 was strongly correlated with that of YAP1 (R = 0.43, *P* < 0.001) in CRC tissues. The expression level of *miR-124-3p* was significantly reduced in human CRC tissues and was negatively correlated with the expression of *Vimentin* at mRNA levels (Pearson R = -0.3386, *P* < 0.05) (Fig. 7D, E), highlighting that miR-124-3p functions as a tumor suppressive miRNA in CRC tissues. Taken together, these data suggest that CXCL12/CXCR7/β-arr1 biased activation triggers YAP1 nuclear translocation, which contributes to EMT and CRC metastasis by repressing miR-124-3p and miR-188-5p in clinical CRC specimens (Fig. 8).

## Discussion

Increasing evidence has shown that CXCR7 is highly expressed in aggressive colonic carcinoma, playing an important role in CRC progression and metastasis [21, 28]. However, the underlying mechanism remains elusive. After CXCR7 was identified as another novel CXCL12 receptor with higher affinity [1], considerable efforts have been made to investigate its functional role and mechanism. CXCR7 has been initially reported to form heterodimers with CXCR4 to modulate CXCR4-mediated cellular responses [2], but some contradicting results also exist [29, 30]. Recently, CXCR7 has been proposed to transmit a biased signal by recruiting β-arrestins to activate MAPK by binding to chemokine CXCL12 without activating typical G-protein signaling pathways [8]. Ligand binding to CXCR7 results in activation of G Protein-Coupled Receptor Kinase 2 (GRK2) and receptor phosphorylation with β-arr2 recruitment. Moreover, the homodimers of CXCR7 but not heterodimers were verified. Importantly, CXCR7 functions as a β‑arr1‑biased receptor and plays an essential role but not auxiliary role in potentiating cell migration [30]. In the present study, we first demonstrated that CXCL12/CXCR7/β‑arr1 biased signal promoted EMT and CRC metastasis by inducing YAP1 nuclear translocation and consequent repressing the expression of miRNAs through recruiting YY1. These findings highlight the important role of YAP1 nuclear translocation in mediating CXCR7-induced cancer metastasis, which could serve as a potential therapeutic target.

β-arrestins (β-arr1 and β-arr2) serve as scaffolds and adapters in receptor trafficking and signal transduction. They are cytosolic proteins connecting the receptors to various cytoplasmic effector pathways such as MAPK cascades [31]. Highly expressed CXCR7 activated MAPK/ERK signaling through ligand-independent but β-arr2-dependent mechanisms [32]. Recent studies uncovered a novel role of β-arrestins in guiding receptor-mediated extracellular signals from cell membrane and transmitting through the cytoplasm to the nucleus by a complicated signaling network [33, 34]. In the present study, we revealed that CXCL12/CXCR7 biased signal transmitted predominantly through β-arr1 to facilitate YAP1 nuclear translocation. We further confirmed the interaction of YAP1 with β-arr1 by Co-IP assay. These results suggest that β-arr1 could bind to YAP1 and promote the accumulation of YAP1 in the nucleus.

Although β-arr1 and β-arr2 share roughly 70% sequence identity and function similarly in GPCR signaling, β-arr1 tends to shuttle between the cytoplasm and the nucleus at steady state, while β-arr2 is excluded from the nucleus due to the presence of a Nuclear Export Signal (NES) [35]. Importantly, recent findings shed light on the role of scaffold protein β-arr1 in mediating the interplay between endothelin-1 receptor (ET-1R) and the Hippo signaling pathway [36]. Other findings also display the function of β-arr1 as a cytoplasm-nucleus messenger in GPCR signaling and elucidate an epigenetic mechanism for transcriptional regulation stimulated by delta-opioid receptor [37]. In this study, we found that β-arr1, interacted with YAP1 in the cytoplasm at early stage of CXCL12 stimulation, triggering YAP1 nuclear translocation and recruiting YY1 to the YAP-TEAD transcriptional complex. In the canonical Hippo pathway, LATS1/2 kinases phosphorylate YAP1, thereby suppressing its nuclear translocation and transcriptional activity. It is possible that CXCL12/CXCR7/β-arr1 biased signal affects the regulation of phosphorylation of YAP1 and further studies on the mechanism are warranted.

EMT, a crucial process in which cells lose their epithelial features and acquire mesenchymal characteristics, is associated with migration, invasion and metastasis of cancer cells [38]. During EMT, epithelial cells gradually lose the expression of E‐cadherin, while concomitantly increasing the expression of mesenchymal markers Vimentin. EMT can be induced through the upregulation of transcriptional regulators such as SNAI1 and ZEB1 [39]. In this study, we found that activation of CXCL12/CXCR7 axis upregulated the expression of EMT markers as shown by increased expression of Vimentin and ZEB1. TCGA datasets further revealed a marked positive correlation of the expression of CXCR7 with EMT markers like Vimentin, ZEB1 and SNAI1. The overexpression of Vimentin in cancer correlates well with increased tumor growth, invasion and poor prognosis and it has gained much importance as a marker for EMT [40]. Besides, highly expressed ZEB1 plays an important role in cancer transformation and EMT [41]. Furthermore, we characterized the regulation of Vimentin expression by miR-124-3p at the post-transcriptional level. Luciferase activity assay proved that miR-124-3p could directly bind to 3’UTR of *Vimentin* and therefore repressed its expression. Interestingly, it has been reported that *ZEB1* is also a target gene of miR-124-3p and the upregulation of ZEB1 could promote EMT by acting as the most important EMT-activating transcription factor [42].

DCLK1, a member of the protein kinase superfamily and the double cortin family, is highly expressed in several types of cancers and has recently been identified as a tumor stem cell marker in CRC [6, 43]. Increasing studies have demonstrated that DCLK1 can promote EMT through downregulating several key tumor suppressor microRNAs and activating NF‐κB through the PI3K/Akt pathway [44, 45], indicating targeting DCLK1 may be a therapeutic option for hampering CRC metastasis. In this study, we found that activation of CXCL12/CXCR7 upregulated DCLK1 by repressing miR-188-5p expression, which further promoted EMT and CRC metastasis. Therefore, CXCL12/CXCR7 induced EMT was mainly mediated by the upregulation of Vimentin, ZEB1 and DCLK1 that were targeted by miR-124-3p and miR-188-5p. Noticeably, the concurrent upregulation of other EMT-related transcriptional factors such as SNAI1 was possibly by other miRNAs or genes.

Among the oncogenic drivers activated by GPCR, YAP1 plays a pivotal role in the progression and metastasis of CRC, linking with worse prognosis of cancer patients [46, 47]. YAP1 is a dual function transcription factor and could function as either a transcriptional activator or repressor which depends on the proteins it interacts with. Recently YAP1 has been shown as a potent transcriptional coactivator to form a complex with ZEB1 to promote EMT plasticity [27]. Additionally, YAP1 could also form a transactivation complex with AP-1 and ZEB1 to activate predominantly tumor-promoting genes [26]. YAP1 also functions as a transcriptional repressor to inhibit the transcription of key cell cycle kinase inhibitor p27 [48]. In this study, we identified YAP1 could interact with YY1 to repress the transcription of miR-124-3p and miR-188-5p. As a member of the GLI-Kruppel family of zinc finger DNA binding proteins, YY1 has been identified as a transcriptional repressor and employs multiple mechanisms to achieve the specific repression [49]. It was predicted that YY1 could bind to the promoters of these miRNAs by TransmiR 2.0 program. Furthermore, luciferase activity assay proved that YY1 could indeed bind to the promoters and repress the transcription of these miRNAs.

Increasing findings have unveiled the correlation between miR-124-3p and miR-188-5p with CRC progression, indicating that these miRNAs could serve as potential targets for CRC therapy [50]. Intriguingly, we elucidated the regulatory roles of these miRNAs on EMT. More importantly, miR-124-3p was also significantly downregulated in CRC compared to adjacent normal colon tissues, suggesting miR-124-3p could serve as a potential diagnostic biomarker and therapeutic target in CRC. Our findings were consistent with previous studies [25]. However, we did not find the significant difference of miR-188-5p between CRC and normal tissues, indicating the function of miR-188-5p possibly depends on the role of target genes and cell context.

## Conclusions

In summary, our studies reveal a novel mechanism and clinical significance of CXCL12/CXCR7 biased signal in promoting EMT and invasion in CRC progression. Although GPCR represents the highly druggable targets, some signaling downstream of a GPCR may be responsible for drug adverse effects. Biased signals hold great promise to become next-generation GPCR drugs with less side effects due to their potential to preferentially activate desired signaling pathways [51]. Targeting CXCL12/CXCR7 biased signal would open a new avenue for improving drug design to achieve higher efficacy and selectivity. We elucidated that biased activation of CXCL12/CXCR7/β-arr1 promoted EMT by repressing miRNAs (miR-124-3p and miR-188-5p) through YAP1 nuclear translocation and recruitment of YY1. More importantly, YAP1 inhibitors attenuated AOM/DSS-induced inflammatory colonic adenocarcinoma in *Villin-CXCR7* transgenic mice as well as impairing distant metastasis of HCT116^LV-CXCR7^ tumor xenografts in nude mice. These findings provide new insights into the nuclear YAP1 in mediating EMT and invasion, highlighting the potential of targeting YAP1 nuclear translocation in hampering CXCL12/CXCR7 biased signal-induced metastasis of CRC.

## Materials and methods

### Cell culture and transfection

Human colorectal adenocarcinoma cell lines HCT116, HT29 and SW620 were purchased from the American Type Culture Collection (ATCC) and cultured in RPMI 1640 medium (Corning, USA) containing 10% fetal bovine serum (FBS) (Biological Industries, Israel) and 1% penicillin–streptomycin (Gibco, USA) at 37 °C in a humid atmosphere (5% CO_2_).

To determine the roles of miR-124-3p and miR-188-5p, cells were transfected with 100 nM of these miRNA mimics or inhibitors (GenePharma, China) using Lipofectamine 2000 (Invitrogen, USA) and Opti-MEM medium (Gibco, USA) for 48 h according to manufacturer’s instructions. The sequences for miR-124-3p and miR-188-5p mimics and inhibitors were listed in Additional file 2: Table S3. Silencing of YAP1, YY1 and β-arr1 were performed by using YAP1, YY1 and β-arr1 siRNAs (GenePharma, China). The sequences for siRNAs were listed in Additional file 2: Table S3. To overexpress YAP1 and YY1 in cancer cells, the cells were transfected with plasmids of Flag-YAP-5SA (#27371, Addgene, USA) and HA-YY1 (#104395, Addgene, USA) by using lipofectamine 2000 (Invitrogen, USA). The CRC cells were infected with lentivirus expressing CXCR7 (LV-CXCR7) and vector control HBLV-CMV-MCS-3flag-EF1-LUC-T2A-PURO (HanBio Biotechnology, China) and screened by puromycin (10 µg/ml) to construct the stable cell lines. Silencing of CXCR7 were performed by using CXCR7 siRNAs (GenePharma, China). The sequences for CXCR7 siRNAs were listed in Additional file 2: Table S3. CXCL12 (SDF-1α) was used at 100 ng/ml and was purchased from PeproTech (USA). AMD3100 was used at 2 μM and was purchased from MedChemExpress LLC (USA).

### Transcriptome sequencings of mRNA and miRNA

Total RNA was extracted from HCT116 cells infected with vector control and LV-CXCR7 (HanBio Biotechnology, China) using Trizol reagent (Invitrogen, USA). RNA integrity and concentration were evaluated by Bioanalyzer 2100 system (Agilent, USA). Library construction and transcriptome sequencing were performed on Hiseq 4000 sequencer and miRNA sequencing was performed on BGISEQ-500 platform by Beijing Genomics Institute (China). Raw reads were filtered using SOAPnuke software and mapped to the human reference genome hg38 using HISAT and Bowtie2 software. Differentially expressed genes (fold change ≥ 1.5 with *P* < 0.05) were analyzed using DESeq2.

### Immunoblotting and co-immunoprecipitation (Co-IP)

Colon tissues and cell pellets were lysed in RIPA buffer (Beyotime, China) supplemented with complete protease inhibitor mixture (Roche Pharmaceuticals, Switzerland) and 1 mM PMSF (Sigma, USA). Nuclear and Cytoplasmic Protein Extraction Kit (Beyotime, China) was used to separate cytoplasmic and nuclear fractions. Protein content of the extracts was determined using BCA Protein Assay Kit (Beyotime, China). Equal amounts of protein (30 µg) were resolved by SDS/PAGE and transferred to PVDF membranes (Millipore, USA). The membranes were blocked and incubated overnight at 4 °C with primary antibodies with 1:1000 dilution against CXCR7 (ab72100) (Abcam, UK), E-cadherin (3195), N-cadherin (13116), Vimentin (5741) (Cell Signaling Technology, USA), YAP1 (13584-1-AP), YY1 (22156-1-AP), β-arr1 (15361-1-AP), β-arr2 (10171-1-AP), DCLK1 (21699-1-AP), Vimentin (10366-1-AP), ZEB1 (21544-1-AP), SNAI1 (13099-1-AP), FLAG tag (20543-1-AP) and HA tag (51064-2-AP) (Proteintech, USA) followed by HRP-conjugated secondary antibodies (7074 and 7076) (Cell Signaling Technology, USA) at a 1:3000 dilution. Densitometric analyses of the bands were normalized with β-actin (66009-1-Ig), GAPDH (60004-1-Ig) (Proteintech, USA), Histone H3 (4499) and Lamin B1 (13435) (Cell Signaling Technology, USA) functioning as loading controls.

Co-IP was carried out using BeaverBeads protein A/G Immunoprecipitation Kit (BEAVER, China) according to the manufacturer’s instructions. Briefly, after pretreatment with binding buffer, 50 µl of protein A/G magnetic beads was incubated with 3 µg of primary antibodies or normal anti-rabbit IgG (2729) (Cell Signaling Technology, USA) at room temperature for 15 min. After washing, the antibody-conjugated protein A/G magnetic beads were incubated with protein lysates at 4 °C overnight. After elution, the immune complexes were subjected to Western blot. Immunoreactive products were visualized using Fluorchem FC3 system (ProteinSimple, USA) by chemiluminescence solution (Millipore, USA) and quantified by using NIH image program (Image J).

### Immunofluorescence staining

Cells stimulated with 100 ng/ml CXCL12 (PeproTech, USA) with or without AMD3100 (MedChemExpress LLC, USA) were fixed in 4% Paraformaldehyde fix solution (Beyotime, China) for 20 min at room temperature. Cells were then washed with PBS, permeabilized in 0.5% Triton™ X-100 (Sigama, USA) in PBS for 10 min and blocked in 5% BSA for 60 min at room temperature. After cells were incubated at 4 °C overnight with anti-YAP1 (1:250, 13584-1-AP) or (1:250, CL594-66900), anti-YY1 (1:200, 22156-1-AP), anti-β-arr1 (1:150, 15361-1-AP) or anti-β-arr2 (1:150, 10171-1-AP) (Proteintech, USA). The next day, Alexa Fluor® 488 donkey anti-rabbit secondary antibodies (1:1000, A21206, Invitrogen, USA) was added to incubate for 1 h at room temperature. DAPI (Beyotime, China) was used for nuclear counterstain for 5 min. Images of representative cells for each labeling condition were captured at × 64 magnitudes under confocal laser imaging system (TCS SP8, Leica, Germany).

### RT-qPCR analysis

Total RNA was reverse-transcribed by the miScript reverse transcription kit (Qiagen, USA) according to the manufacturer’s protocol. MiScript SYBR Green PCR kit (Qiagen, USA), miR-124-3p and miR-188-5p specific primers were used to determine the expression of mature miRNAs. RUN6B was used as an internal control. To determine the mRNA expression of *CXCR7*, *Vimentin*, *DCLK1* and *E-cadherin*, RNA extracts were reverse-transcribed into cDNA using ReverTra Ace qPCR RT Kit (TOYOBO, Japan). RT-qPCR was carried out using SYBR Green Realtime PCR Master Mix (TOYOBO, Japan) in the 7500 Fast Real-Time PCR System (Applied Biosystems, USA). The relative gene expression was calculated using 2^−ΔΔ*C*t^method as described previously [52]. The sequences of primers for PCR are listed in Additional file 2: Table S4.

### Immunohistochemistry

Colorectal tissues were fixed in 4% polyformaldehyde followed by paraffin embedding and then the sections were deparaffinized, rehydrated and immersed in 10 mM citrate buffer for heat-induced antigen retrieval. The tissues were stained with antibodies against CXCR7 (ab72100) (Abcam, UK), Vimentin (5741) (Cell Signaling Technology, USA) and YAP1 (13584-1-AP) (Proteintech, USA) (1:200 dilution) in accordance with manufacturer’s suggestions. The staining results were scanned using a digital slide scanning system (Pannoramic Scan, MedicalEXPO, France) and semi-quantified of mean density (IOD/area) by Image-Pro Plus 6.0 software (IPP).

### Luciferase reporter gene assay

To confirm that *DCLK1* and *Vimentin* were the target genes of miR-188-5p and miR-124-3p respectively, the 3’UTR of *DCLK1* and *Vimentin* containing miRNA binding sites were synthesized and digested by *Xba* I and cloned into GV272 vector (GeneChem, China) to make the luciferase constructs. Wild type and mutant inserts were confirmed by sequencing. GV272 empty vector was used as control (Con081). To investigate the effect of transcriptional factor YY1 on the promoter activities of *miR-188-5p* and *miR-124-3p*, the promoter sequences of these miRNAs containing YY1 binding sites were digested by *Nhe* I and *Hind* III and cloned into pGL3-basic plasmid (Promega, USA).

The luciferase reporter assay was performed as previously described [52]. Briefly, HCT116 cells were co-transfected with wild type or mutant *DCLK1*-3’UTR-Luc/*Vimentin*-3’UTR-Luc firefly luciferase constructs (100 ng) and 40 nM miR-188-5p/miR-124-3p mimics using lipofectamine 2000 reagent. 2 ng of pRL-SV40 plasmid was transfected to monitor transfection efficiency. Luciferase activity was determined by a dual-luciferase reporter assay system (Promega, USA). All assays were performed in triplicates for twice.

### Animal models

*Villin-CXCR7* transgenic mice (*Villin-CXCR7*) overexpressing CXCR7 in intestinal epithelial cells (IEC) were generated by Cyagen Biosciences Inc. (China). To establish azoxymethane (AOM)/ dextran sodium sulfate (DSS)-induced inflammatory colonic adenocarcinoma, wild type C57BL/6 mice and *Villin-CXCR7* mice (aged at 8 weeks, n = 5) were treated by a single intraperitoneal injection of AOM (10 mg/kg, Sigma, USA) and subsequent oral administration of 1% DSS (MP Biomedicals, USA) in drinking water ad libitum for 7 consecutive days and then returned to normal drinking for 14 days. *Villin-CXCR7* mice were injected intraperitoneally daily with YAP1 inhibitor Verteporfin (10 mg/kg, MedChemExpress LLC, USA) in the vehicle (40% PEG300, 5% Tween 80 and 45% normal saline) or vehicle control for 8 consecutive weeks. After 3 cycles of DSS treatment, these mice were euthanized and their colons were excised and opened longitudinally to evaluate the number and size of tumors with a caliper.

Six-week-old female athymic *Balb/c-nu/nu* mice were purchased from Charles River Laboratories (China) and maintained in a specific pathogen-free environment. 2 × 10^6^ HCT116 cells infected with LV-CXCR7 and vector control were injected into nude mice via tail veins. The mice injected with HCT116^LV−CXCR7^ cells were administered daily with YAP1 inhibitor Verteporfin (10 mg/kg, MedChemExpress LLC, USA) or solvent control. After 3 weeks, the mice were sacrificed and their livers and lungs were removed for histologic examination. All animal experiments were approved by the Institutional Animal Care and Use Committee of Capital Medical University. The ethics number was AEEI-2021-194.

### Clinical specimens

Human CRC tissue specimens and adjacent normal mucosa were obtained from XuanWu Hospital of Capital Medical University (Beijing, China) and were confirmed by pathological analysis. The experiment was approved by the Institutional Review Board of Capital Medical University. The informed consents were obtained from all patients. The study methodologies conformed to the standards set by the Declaration of Helsinki.

### Bioinformatics

Gene Expression Profiling Interactive Analysis (GEPIA) (http://GEPIA.cancer-pku.cn/index.html) was a web server to analyze gene sequencing data from the cancer genome atlas (TCGA) and Genotype-Tissue Expression (GTEx). GEPIA was used to perform gene expression spearman correlation analysis of CXCR7 and Vimentin or DCLK1 and other EMT-related genes in CRC patients. The association of the expression of Vimentin and DCLK1 with overall survival was also analyzed by GEPIA in gastrointestinal cancer (stomach, colon and rectum adenocarcinomas combined). The patients were divided with high and low gene expression levels using the median and log-rank *P* value was shown.

### Statistical analysis

All data were presented as mean ± SD and statistical data were analyzed using SPSS 20.0 or graphpad prism 8.0. Statistical differences among multiple groups were evaluated by one-way analysis of variance (ANOVA) followed by Dunnett (multiple comparisons to the same control) post hoc tests. Student’s *t* test was used to compare differences between two groups. The Pearson correlation was used to evaluate potential correlations between miR-124-3p and Vimentin expression in paired CRC tissues. A *P* < 0.05 was considered to indicate statistical significance.

## Supplementary Information

**Additional file 1: Fig. S1.** **A**, **B** RT-qPCR and Western blot analysis of the expression of CXCR7 at mRNA and protein levels in CRC cells. **Fig. S2.** Transwell assay was performed in HCT116^CXCR7^ and SW620^CXCR7^ cells compared with controls. Migrating cells were quantified. **P < 0.01. ***P < 0.001. **Fig. S3.** The correlations of CXCR7 (ACKR3) with ZEB1 and SNAI1 were determined in CRC tissues by Gene Expression Profiling Interactive Analysis (GEPIA). **Fig. S4.** **A**, **B** RT-qPCR analysis of the levels of *miR-124-3p* and *miR-188-5p* in HCT116, HT29 and SW620 cells transfected with these miRNA mimics (124m, 188m) and inhibitors (124i, 188i). **Fig. S5. A**, **B** Western blot analysis of the expression levels of YAP1, DCLK1 and Vimentin in HCT116 and SW620 cells transfected Flag-YAP-5SA compared with vector control. Anti-Flag antibodies were used to indicate the overexpression of Flag-YAP-5SA plasmid in CRC cells. **Fig. S6.** IF analysis of YAP1 nuclear translocation in HCT116 cells treated with CXCL12 (100 ng/ml) for different time course as indicated. YAP1 was labeled with Alexa Fluor® 488 donkey anti-rabbit secondary antibody. Nuclei were visualized with DAPI. Scale bars, 50 µm. **Fig. S7.** Western blot analysis of the expression levels of β-arrestin1 in HCT116 nuclear extracts stimulated by CXCL12 (100 ng/ml) for different time course as indicated. GAPDH and Histone H3 were used as cytoplasmic and nuclear loading control, respectively. **Fig. S8.** Transcriptional factor YY1 was predicted to bind to promoters of miR-124-3p and miR-188-5p by TransmiR 2.0 database. **Fig. S9. **Western blot analysis of the expression levels of YY1, DCLK1 and Vimentin in HCT116 and SW620 cells transfected with vector control and HA-YY1 plasmid. HA-tag was used to indicate the overexpression of YY1 in CRC cells. **Fig. S10.** The correlations of YAP1 with ZEB1 and SNAI1 were determined in CRC tissues by Gene Expression Profiling Interactive Analysis (GEPIA).

**Additional file 2: Table S1.** The significantly upregulated genes in HCT116^LV-CXCR7^ vs. HCT116^Control^. **Table S2. **The significantly upregulated and downregulated miRNAs in HCT116^LV-CXCR7^ vs. HCT116^Control^. **Table S3.** The sequences of siRNAs and miRNAs. **Table S4.** The sequences of primers for RT-qPCR.

## Data Availability

The data that support the findings of this study are available from the corresponding author (xyu@ccmu.edu.cn) upon reasonable request. Additional data are available as Additional files. The raw data from the analysis have been submitted to Sequence Read Archive (SRA) data with BioProject accession number PRJNA784641.

## Abbreviations

CRC: Colorectal cancer
CXCR4: Chemokine CXC motif receptor 4
CXCR7: Chemokine CXC motif receptor 7
CXCL12: Chemokine CXC motif ligand 12
CSCs: Cancer stem cells
DCLK1: Doublecortin-like kinase 1
EMT: Epithelial-to-mesenchymal transition
GPCR: G protein-coupled receptor
NC: Negative control
siRNA: Small interfering RNA
TAZ: Transcriptional coactivator with PDZ-binding motif
TEAD: Transcriptional enhanced associate domain
WT: Wild-type
YAP: Yes-associated protein
YY1: Yin Yang 1
