# Supplementary figures and images for: CXCL12/CXCR7/β-arrestin1 biased signal promotes epithelial-to-mesenchymal transition of colorectal cancer by repressing miRNAs through YAP1 nuclear translocation
Source: Cell Biosci. 2022 Oct 9;12:171. doi: 10.1186/s13578-022-00908-1 (PMC9549625; doi:10.1186/s13578-022-00908-1)

Fig. S1

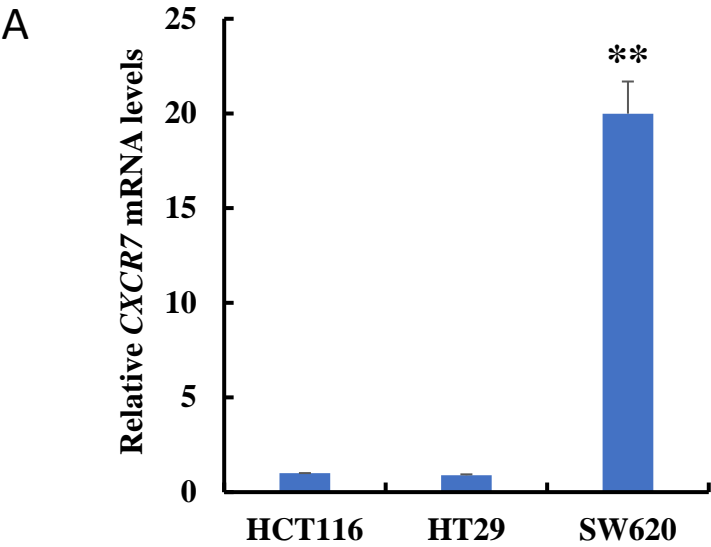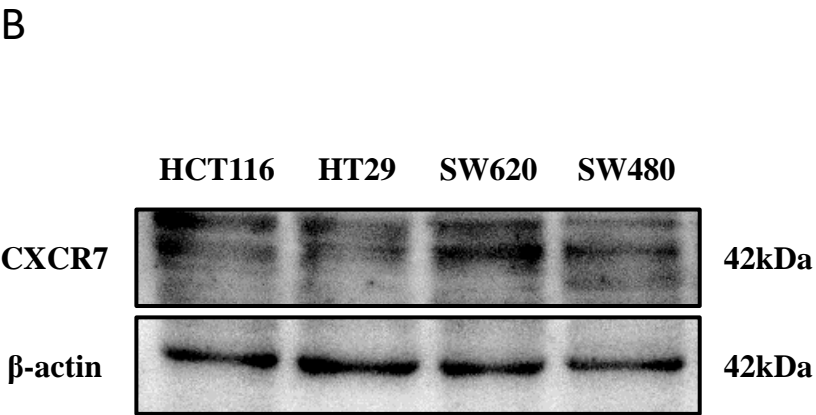

Fig. S2

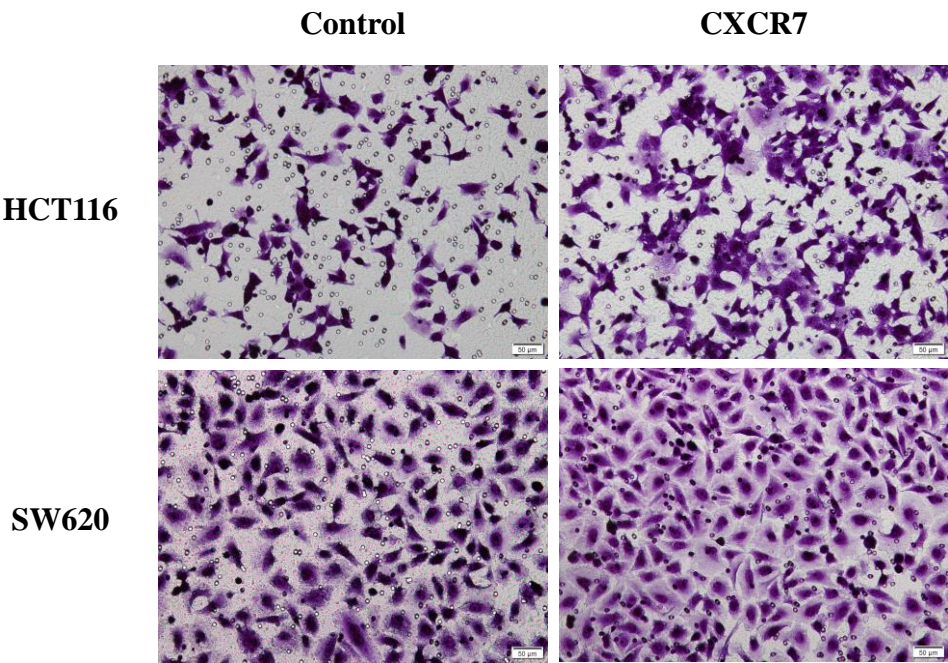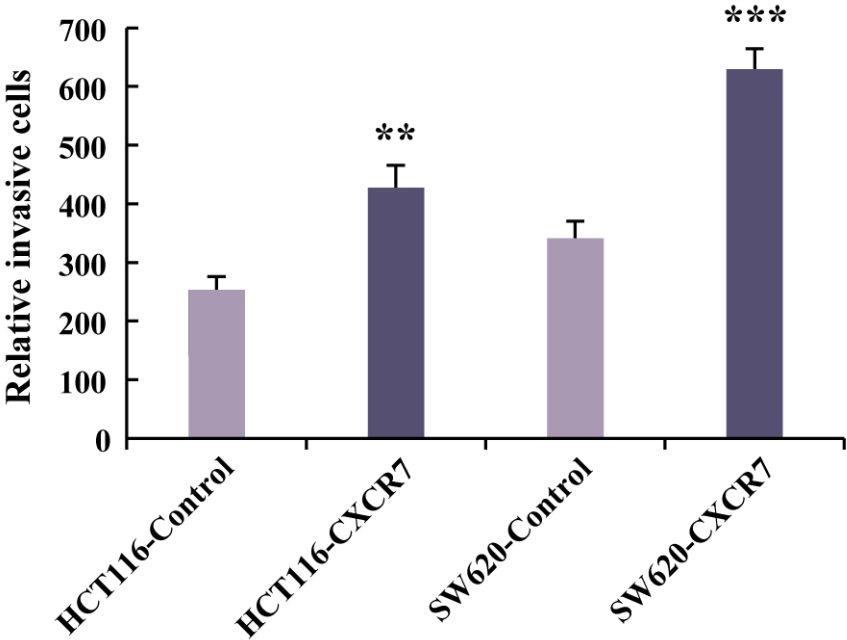

Fig. S3

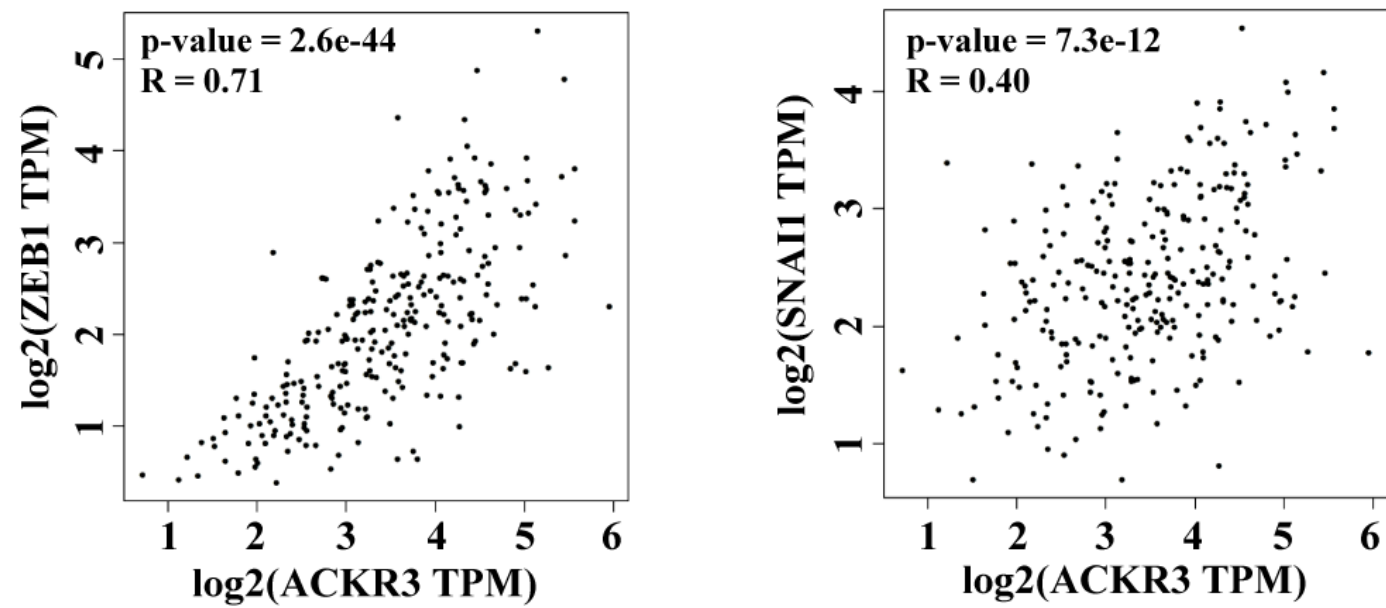

Fig. S4

A

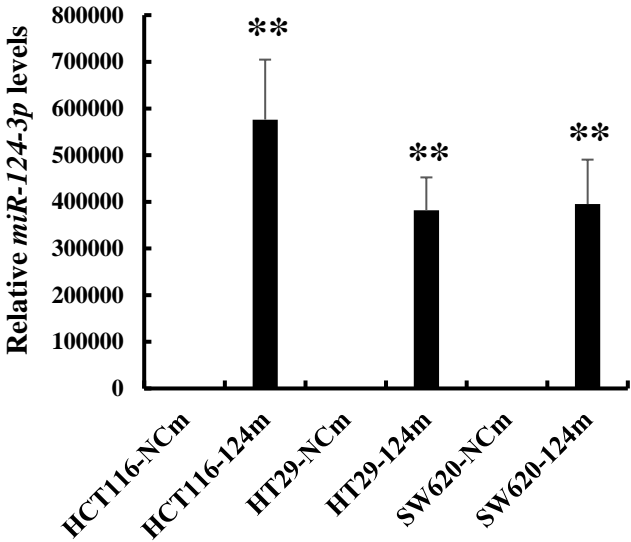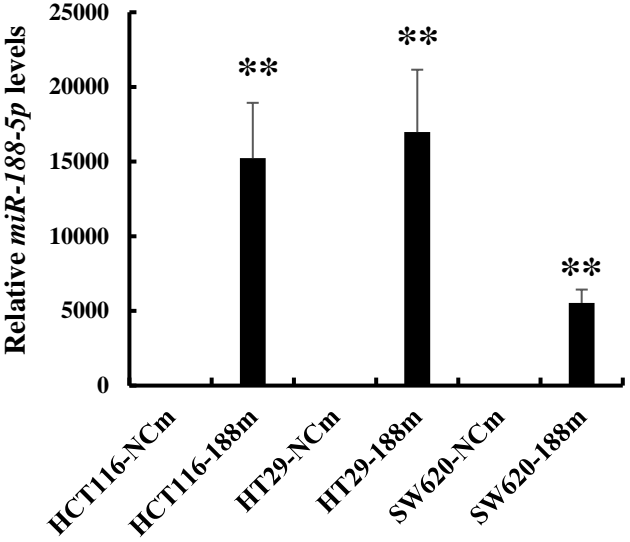

B

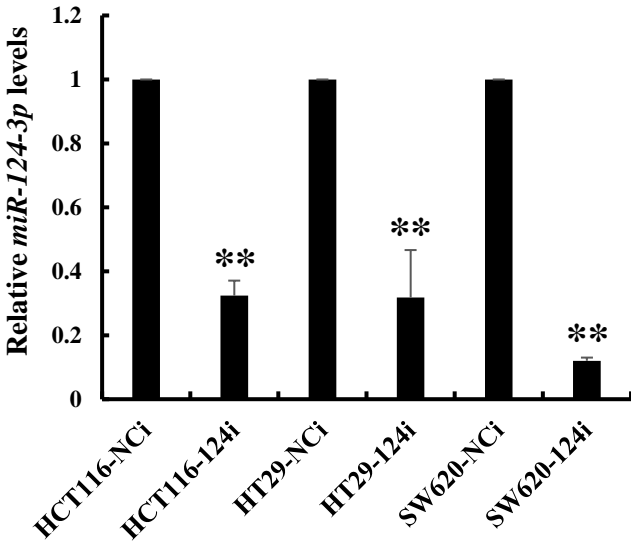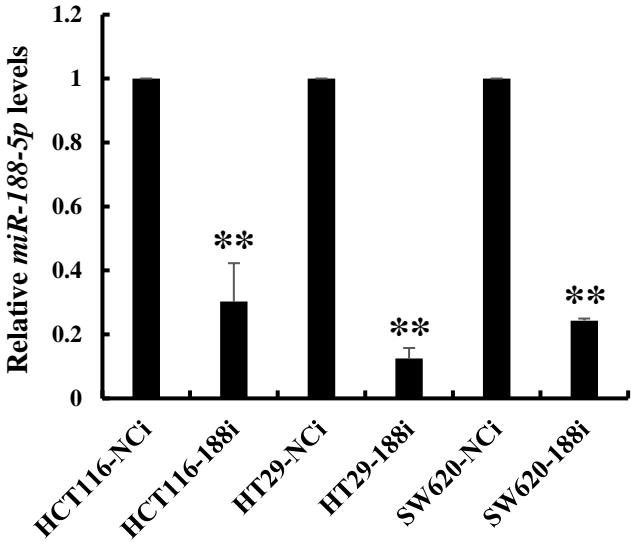

Fig. S5

A

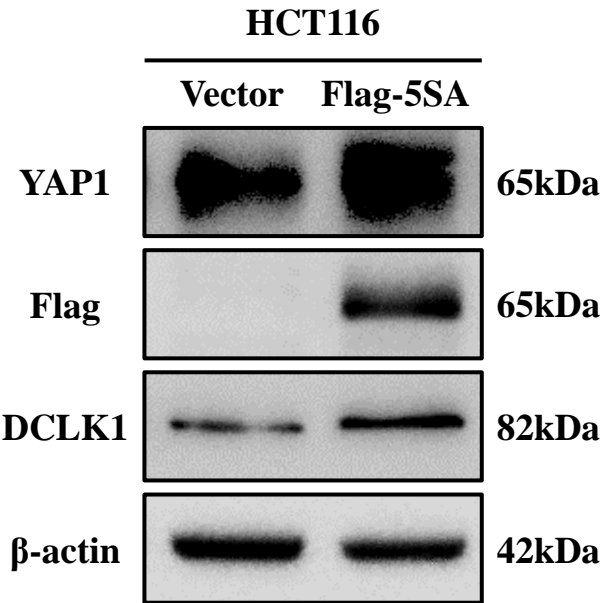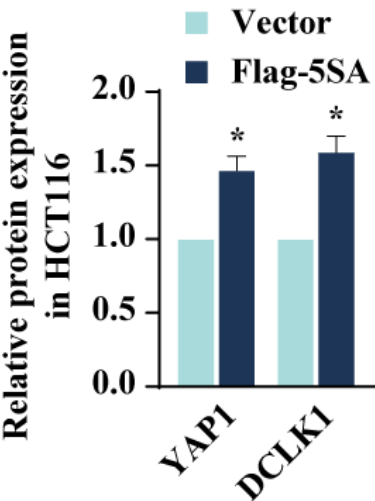

B

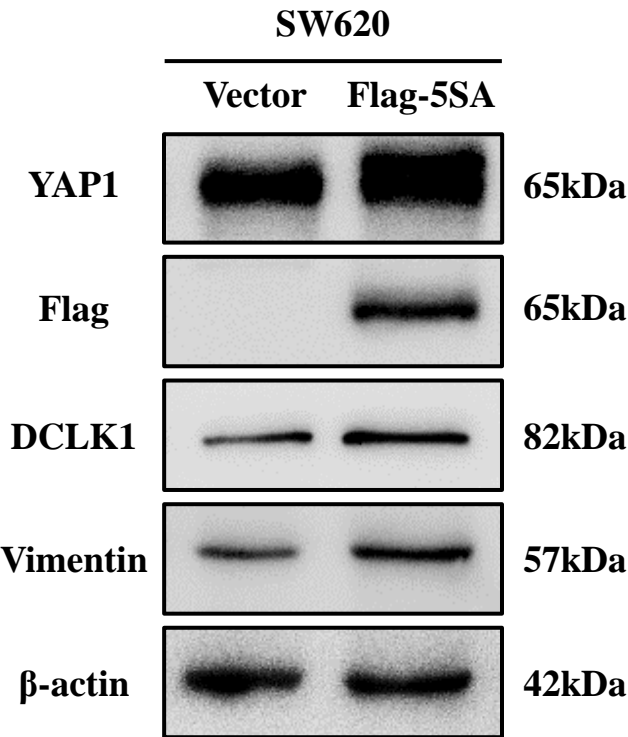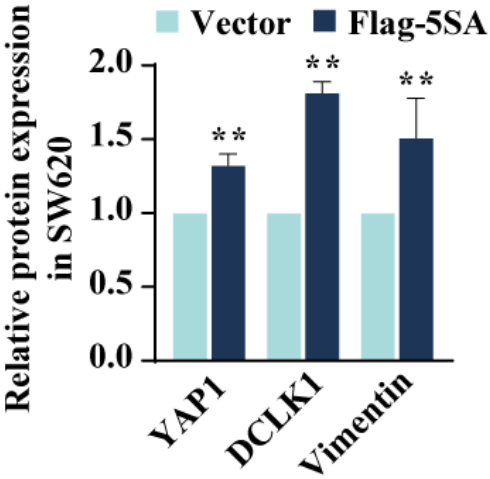

Fig. S6

HCT116-CXCL12

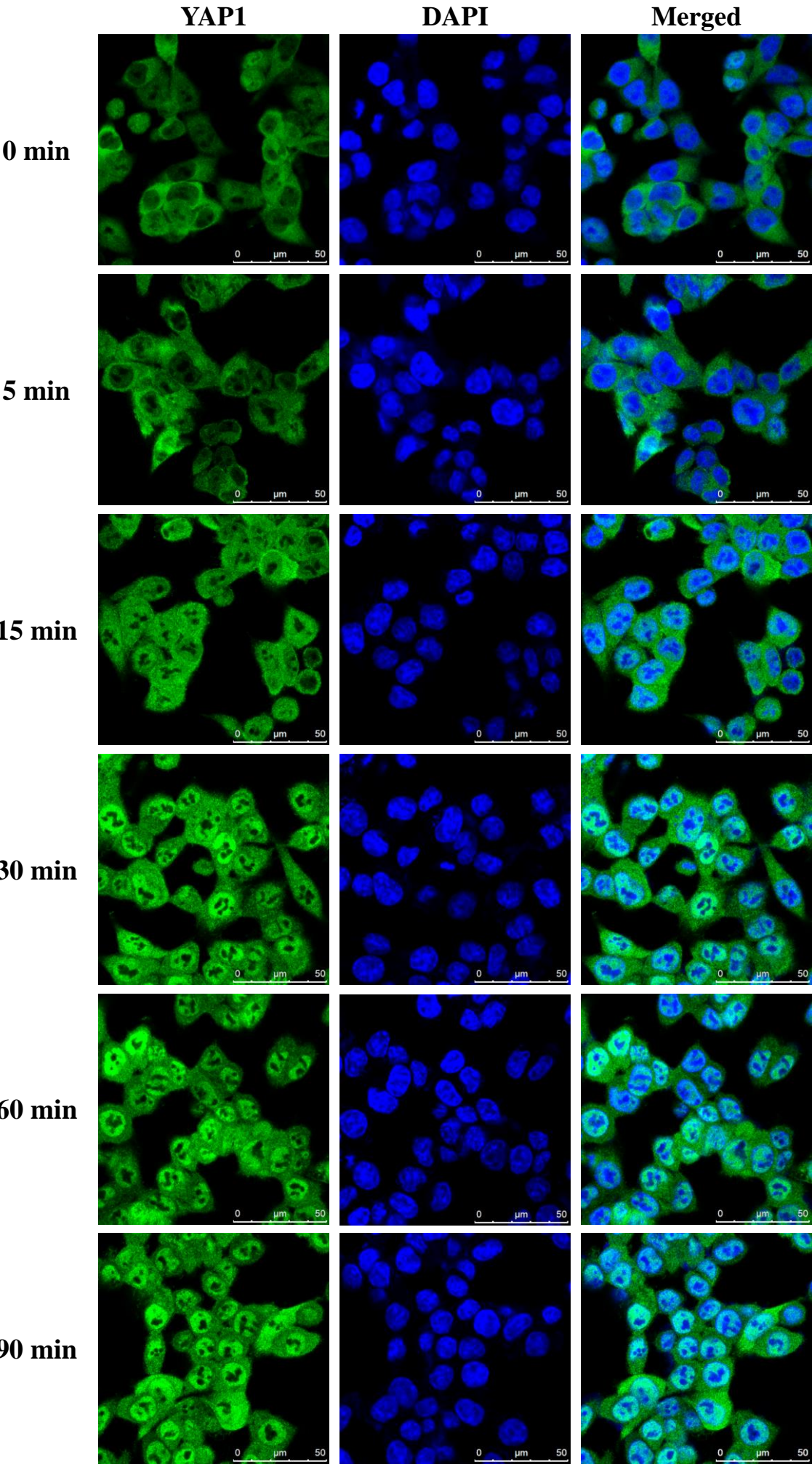

Fig. S7

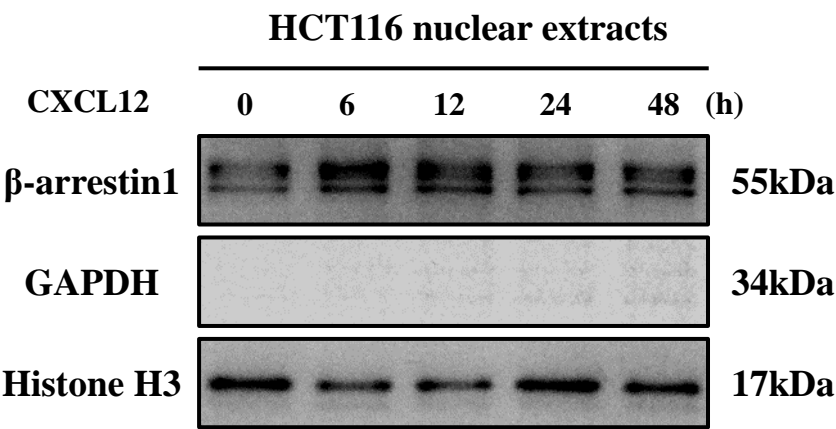

Fig. S8

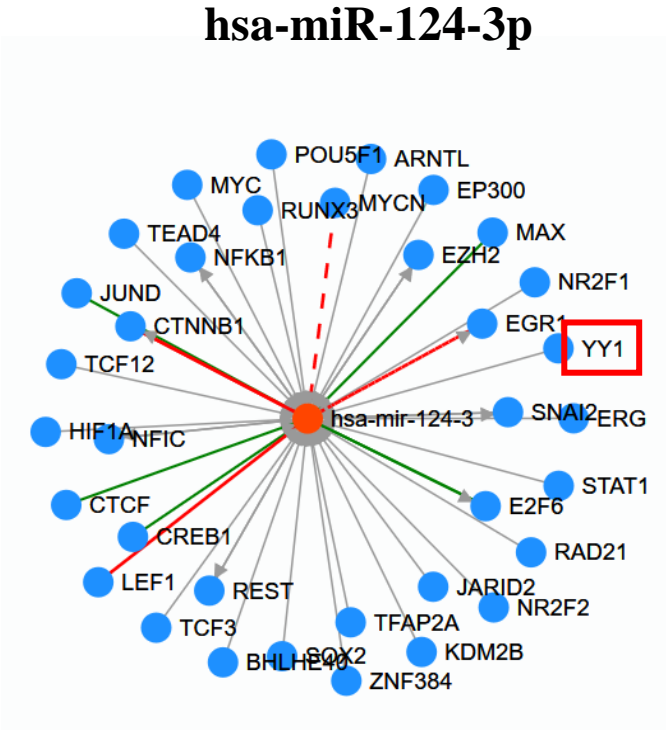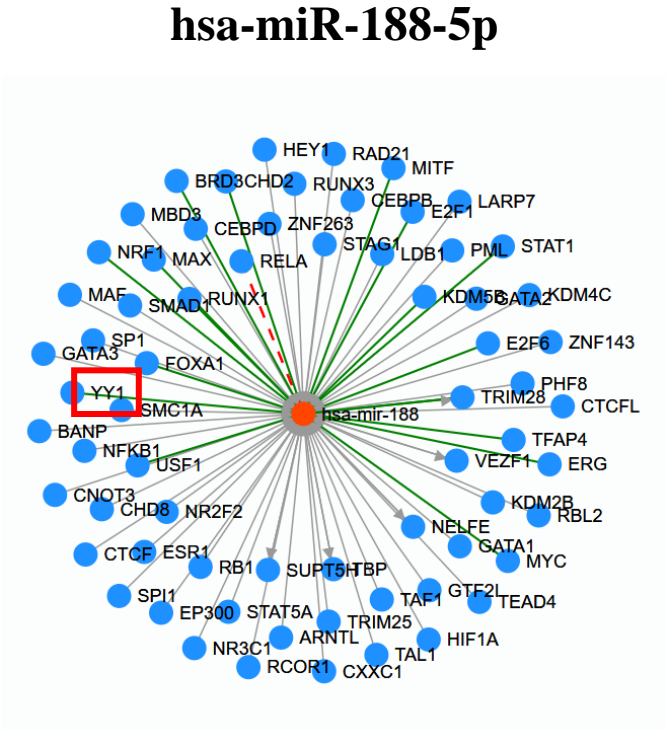

Predicted by TransmiR v2.0 database

Fig. S9

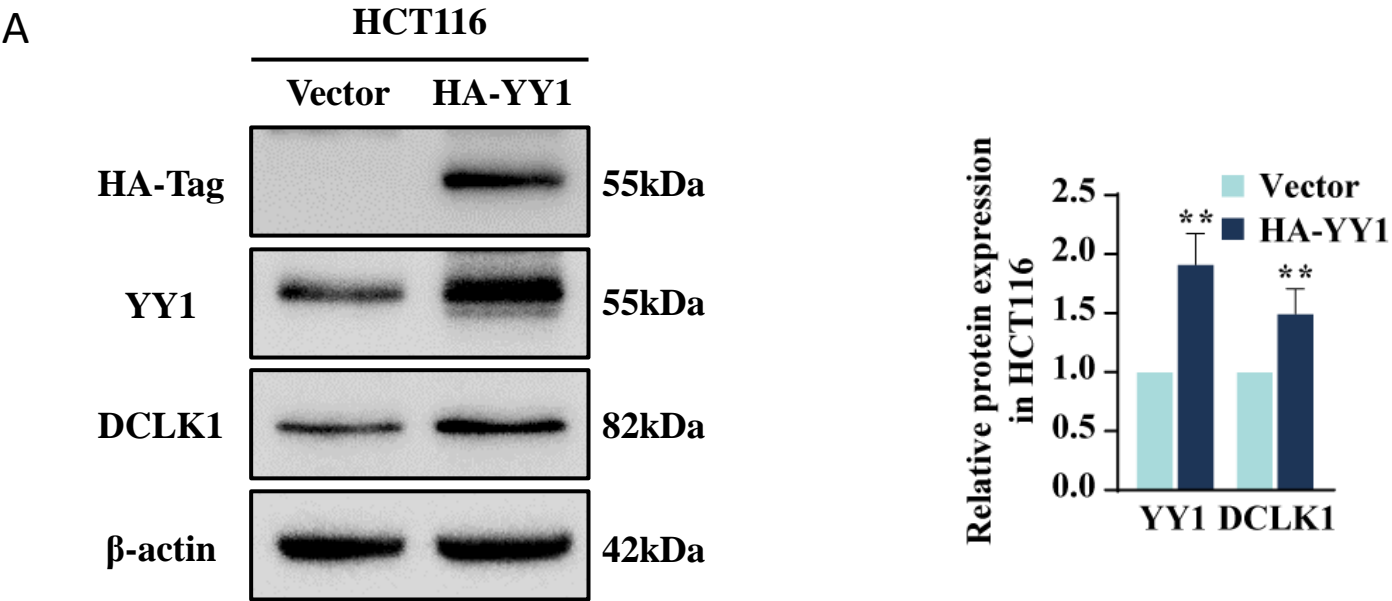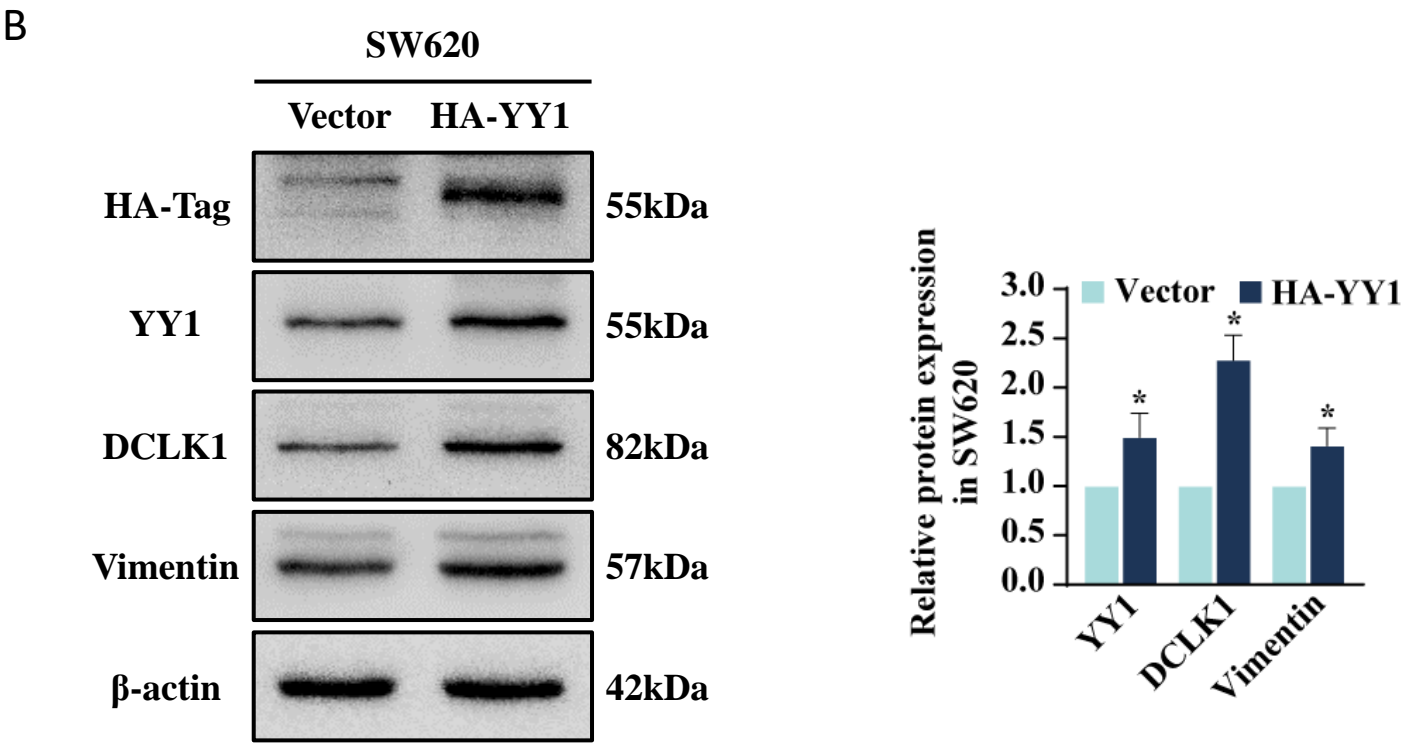

Fig. S10

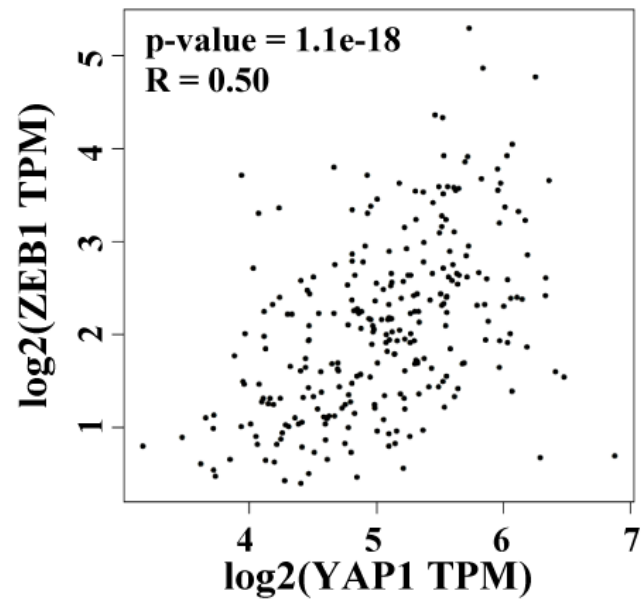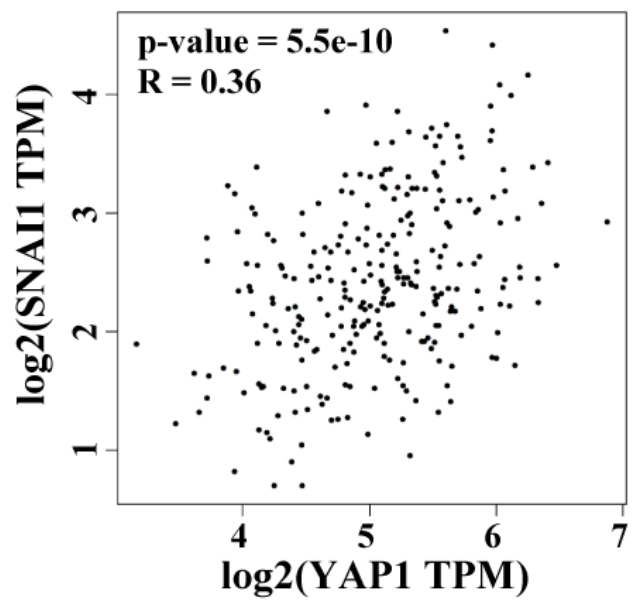

Supplement: Supplementary file 1 — Additional file 1: Fig. S1. A, B RT-qPCR and Western blot analysis of the expression of CXCR7 at mRNA and protein levels in CRC cells. Fig. S2. Transwell assay was performed in HCT116CXCR7 and SW620CXCR7 cells compared with controls. Migrating cells were quantified. **P < 0.01. ***P < 0.001. Fig. S3. The correlations of CXCR7 (ACKR3) with ZEB1 and SNAI1 were determined in CRC tissues by Gene Expression Profiling Interactive Analysis (GEPIA). Fig. S4. A, B RT-qPCR analysis of the levels of miR-124-3p and miR-188-5p in HCT116, HT29 and SW620 cells transfected with these miRNA mimics (124m, 188m) and inhibitors (124i, 188i). Fig. S5. A, B Western blot analysis of the expression levels of YAP1, DCLK1 and Vimentin in HCT116 and SW620 cells transfected Flag-YAP-5SA compared with vector control. Anti-Flag antibodies were used to indicate the overexpression of Flag-YAP-5SA plasmid in CRC cells. Fig. S6. IF analysis of YAP1 nuclear translocation in HCT116 cells treated with CXCL12 (100 ng/ml) for different time course as indicated. YAP1 was labeled with Alexa Fluor® 488 donkey anti-rabbit secondary antibody. Nuclei were visualized with DAPI. Scale bars, 50 µm. Fig. S7. Western blot analysis of the expression levels of β-arrestin1 in HCT116 nuclear extracts stimulated by CXCL12 (100 ng/ml) for different time course as indicated. GAPDH and Histone H3 were used as cytoplasmic and nuclear loading control, respectively. Fig. S8. Transcriptional factor YY1 was predicted to bind to promoters of miR-124-3p and miR-188-5p by TransmiR 2.0 database. Fig. S9. Western blot analysis of the expression levels of YY1, DCLK1 and Vimentin in HCT116 and SW620 cells transfected with vector control and HA-YY1 plasmid. HA-tag was used to indicate the overexpression of YY1 in CRC cells. Fig. S10. The correlations of YAP1 with ZEB1 and SNAI1 were determined in CRC tissues by Gene Expression Profiling Interactive Analysis (GEPIA). [file 13578_2022_908_MOESM1_ESM.zip › 13578_2022_908_MOESM1_ESM/Supplementary Figures revision - Copy.pdf]
