# Supplementary material for: CXCL12/CXCR7/β-arrestin1 biased signal promotes epithelial-to-mesenchymal transition of colorectal cancer by repressing miRNAs through YAP1 nuclear translocation
Source: Cell Biosci. 2022 Oct 9;12:171. doi: 10.1186/s13578-022-00908-1 (PMC9549625; doi:10.1186/s13578-022-00908-1)
Supplement: Supplementary file 2 — Additional file 2: Table S1. The significantly upregulated genes in HCT116LV-CXCR7 vs. HCT116Control. Table S2. The significantly upregulated and downregulated miRNAs in HCT116LV-CXCR7 vs. HCT116Control. Table S3. The sequences of siRNAs and miRNAs. Table S4. The sequences of primers for RT-qPCR. [file 13578_2022_908_MOESM2_ESM.docx]

**Supplementary Table 1. The significantly upregulated genes in HCT116^LV-CXCR7^ *v.s.* HCT116^Control^**

| Gene ID | Gene Symbol | log_2_ (CXCR7/Control) | P value (CXCR7/Control) |
| --- | --- | --- | --- |
| 107987254 | *LOC107987254* | 7.91886 | 0 |
| 1241 | *LTB4R* | 6.52356 | 0 |
| 445329 | *SULT1A4* | 4.49185 | 0 |
| 79025 | *FNDC11* | 3.558 | 0 |
| 646498 | *C3orf84* | 3.45943 | 0 |
| 3553 | *IL1B* | 3.36737 | 0 |
| 1018 | *CDK3* | 2.89077 | 1.00E-05 |
| 345275 | *HSD17B13* | 2.51785 | 0 |
| 84752 | *B3GNT9* | 2.45943 | 0 |
| 126272 | *EID2B* | 2.42626 | 1.00E-05 |
| 11156 | *PTP4A3* | 2.29662 | 0 |
| 54567 | *DLL4* | 2.23704 | 0 |
| 79585 | *CORO7* | 2.07039 | 0 |
| 4112 | *MAGEB1* | 1.91886 | 0 |
| 9278 | *ZBTB22* | 1.87915 | 0 |
| 116441 | *TM4SF18* | 1.84246 | 0 |
| 7431 | *VIM* | 1.83289 | 0 |
| 107986353 | *LOC107986353* | 1.71049 | 0 |
| 51136 | *RNFT1* | 1.69014 | 0 |
| 10379 | *IRF9* | 1.32992 | 0 |
| 3084 | *NRG1* | 1.24961 | 0 |
| 4157 | *MC1R* | 1.1809 | 0 |
| 1415 | *CRYBB2* | 0.98138 | 2.00E-05 |
| 338596 | *ST8SIA6* | 0.9752 | 2.00E-05 |
| 10156 | *RASA4* | 0.95109 | 0 |
| 27345 | *KCNMB4* | 0.82384 | 2.00E-05 |
| 9201 | *DCLK1* | 0.80498 | 0 |
| 10317 | *B3GALT5* | 0.78338 | 0 |
| 119385 | *AGAP11* | 0.75829 | 2.00E-05 |
| 80274 | *SCUBE1* | 0.75707 | 0 |
| 114625 | *ERMAP* | 0.74076 | 2.00E-05 |
| 66035 | *SLC2A11* | 0.739 | 1.00E-05 |
| 9120 | *SLC16A6* | 0.71675 | 0 |
| 79633 | *FAT4* | 0.70555 | 0 |
| 687 | *KLF9* | 0.68549 | 1.00E-05 |
| 6935 | *ZEB1* | 0.5961 | 0 |

**Supplementary Table 2. The significantly upregulated and downregulated miRNAs in HCT116^LV-CXCR7^ *v.s.* HCT116^Control^**

| Gene ID | Gene Symbol | log_2_  (CXCR7/Control) | P value (CXCR7/Control) |
| --- | --- | --- | --- |
| hsa-miR-302d-3p | *hsa-miR-302d-3p* | 13.18302 | 0 |
| hsa-miR-7-5p | *hsa-miR-7-5p* | 6.43825 | 0 |
| hsa-miR-302b-3p | *hsa-miR-302b-3p* | 5.40599 | 0 |
| hsa-miR-155-5p | *hsa-miR-155-5p* | 5.12928 | 0 |
| hsa-miR-30a-3p | *hsa-miR-30a-3p* | 4.2854 | 0 |
| hsa-miR-30a-5p | *hsa-miR-30a-5p* | 3.97728 | 0 |
| hsa-miR-302c-3p | *hsa-miR-302c-3p* | 3.58496 | 0 |
| hsa-miR-302a-3p | *hsa-miR-302a-3p* | 3.56768 | 0 |
| hsa-miR-624-3p | *hsa-miR-624-3p* | 3.32193 | 6.00E-04 |
| hsa-miR-339-5p | *hsa-miR-339-5p* | 2.81816 | 0 |
| hsa-miR-146a-5p | *hsa-miR-146a-5p* | 2.71172 | 0 |
| hsa-miR-20b-5p | *hsa-miR-20b-5p* | 2.53605 | 4.00E-04 |
| hsa-miR-363-3p | *hsa-miR-363-3p* | 2.36923 | 0 |
| hsa-miR-664a-3p | *hsa-miR-664a-3p* | 2.02975 | 9.00E-05 |
| hsa-miR-124-3p | *hsa-miR-124-3p* | -1.848 | 9.10E-04 |
| hsa-miR-940 | *hsa-miR-940* | -1.8501 | 0 |
| hsa-miR-188-5p | *hsa-miR-188-5p* | -1.90215 | 0 |
| hsa-miR-451a | *hsa-miR-451a* | -2.36967 | 0 |
| hsa-miR-15a-3p | *hsa-miR-15a-3p* | -2.70044 | 0 |
| hsa-let-7i-3p | *hsa-let-7i-3p* | -2.71621 | 0 |
| hsa-miR-423-5p | *hsa-miR-423-5p* | -3.68182 | 0 |
| hsa-miR-642b-5p | *hsa-miR-642b-5p* | -3.70044 | 0 |
| hsa-miR-188-3p | *hsa-miR-188-3p* | -9.64386 | 0.00119 |

**Supplementary Table 3. The sequences of siRNAs and miRNAs**

| Gene | RNA Sequence (5′ to 3′) | Species |
| --- | --- | --- |
| si-CXCR7 | 5’-cgcucuccuucauuuacauuu-3’  5’-aaauguaaaugaaggagagcg-3’ | *Homo sapiens* |
| si-YAP1-1 | 5’-caccaagcuagauaaagaatt-3’  5’-uucuuuaucuagcuugguggc-3’ | *Homo sapiens* |
| si-YAP1-2 | 5’-agcuucucugcaguugggagcugtt-3’  5’-aacagcucccaacugcagagaagcu-3’ | *Homo sapiens* |
| si-YY1-1 | 5’-gacgacuacauugaacaaatt-3’  5’-uuuguucaauguagucguctt-3’ | *Homo sapiens* |
| si-YY1-2 | 5’-cccaaacaacuggcagaautt-3’  5’-auucugccaguuguuugggtt-3’ | *Homo sapiens* |
| si-β-arrestin1 | 5’-agccuucugcgcggagaautt-3’  5’-auucuccgcgcagaaggcuuu-3’ | *Homo sapiens* |
| si-β-arrestin2 | 5’-ggaccgcaaaguguuugugtt-3’  5’-cacaaacacuuugcgguccuu-3’ | *Homo sapiens* |
| miR-124-3p mimics | 5’-uaaggcacgcggugaaugccaa-3’  5’-ggcauucaccgcgugccuuauu-3’ | *Homo sapiens* |
| miR-188-5p mimics | 5’-caucccuugcaugguggaggg-3’  5’-cuccaccaugcaagggauguu-3’ | *Homo sapiens* |
| miR-124-3p inhibitors | 5’-uuggcauucaccgcgugccuua-3’ | *Homo sapiens* |
| miR-188-5p inhibitors | 5’-cccuccaccaugcaagggaug-3’ | *Homo sapiens* |

**Supplementary Table 4. The sequences of primers for RT-QPCR**

| Gene | Primer Sequence (5′ to 3′) | Species |
| --- | --- | --- |
| *CXCR7* | F: tccatcctgcactacatccc  R: actctggaggcatcgatgag | *Homo sapiens* |
| *E-cadherin* | F: acgcattgccacatacactc  R: agaggttcctggaagagcac | *Homo sapiens* |
| *Vimentin* | F: agatggcccttgacattgag  R: ccagagggagtgaatccaga | *Homo sapiens* |
| *DCLK1* | F: ccatcatcagaagtggcgtg  R: cctgtttcccatccaacgtg | *Homo sapiens* |
